# Structural motifs and intramolecular interactions in non-canonical G-quadruplexes

**DOI:** 10.1039/d0cb00211a

**Published:** 2021-01-22

**Authors:** Jagannath Jana, Swantje Mohr, Yoanes Maria Vianney, Klaus Weisz

**Affiliations:** Institute of Biochemistry, Universität Greifswald Felix-Hausdorff-Str. 4 D-17487 Greifswald Germany weisz@uni-greifswald.de +49 3834 420-4427 +49 3834 420-4426

## Abstract

Guanine(G)-rich DNA or RNA sequences can assemble or intramolecularly fold into G-quadruplexes formed through the stacking of planar G·G·G·G tetrads in the presence of monovalent cations. These secondary nucleic acid structures have convincingly been shown to also exist within a cellular environment exerting important regulatory functions in physiological processes. For identifying nucleic acid segments prone to quadruplex formation, a putative quadruplex sequence motif encompassing closely spaced tracts of three or more guanosines is frequently employed for bioinformatic search algorithms. Depending on the number and type of intervening residues as well as on solution conditions, such sequences may fold into various canonical G4 topologies with continuous G-columns. On the other hand, a growing number of sequences capable of quadruplex formation feature G-deficient guanine tracts, escaping the conservative consensus motif. By folding into non-canonical quadruplex structures, they adopt unique topologies depending on their specific sequence context. These include G-columns with only two guanines, bulges, snapback loops, D- and V-shaped loops as well as interlocked structures. This review focuses on G-quadruplex species carrying such distinct structural motifs. It evaluates characteristic features of their non-conventional scaffold and highlights principles of stabilizing interactions that also allow for their folding into stable G-quadruplex structures.

## Abstract 2

G-rich nucleic acid sequences encompassing G-tracts of varying lengths can fold into different non-canonical G-quadruplexes with distinct structural features.

## Graphical abstract

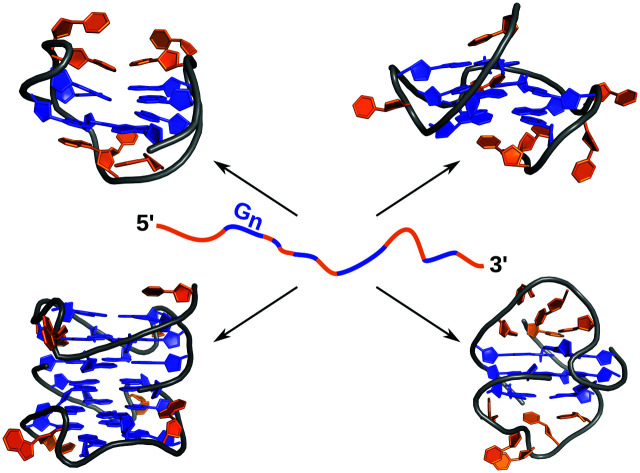

## Introduction

Single-stranded guanine-rich DNA or RNA sequences can fold into intramolecular or intermolecular four-stranded structures called G-quadruplexes (G4s). G4-prone motifs are found in high numbers not only in bacterial and viral, but also in human genomes. Thus, G-rich oligonucleotides derived from genomic sequences like those from oncogene promoters and telomeres have been demonstrated to fold into G-quadruplexes. Through their visualization, compelling evidence for the existence of these non-canonical secondary nucleic acid structures has also been found in cellular environments.^1,2^ Our current understanding of the biological roles of quadruplexes suggests that G4s are involved in gene regulation and telomere maintenance, making genomic quadruplexes promising therapeutic targets.^3^ In this regard, much effort has been devoted during the last decades to searching for G4-stabilizing ligands for pharmaceutical intervention, *e.g.*, for modulating gene expression or telomerase inhibition in cancer cells.^4^ In addition to serving as potential drug targets, synthetic quadruplexes such as the thrombin binding aptamer (TBA) or anti-HIV-1 integrase aptamer constitute an emerging class of therapeutics, binding to various molecules including many pathologically relevant proteins with very high affinity and selectivity.^5,6^ Finally, the increasing use of quadruplexes in supramolecular chemistry as well as in biosensors and nanotechnology as a result of their ability to self-organize into complex two-dimensional networks and long nanowires attests to their enormous potential in medicinal and technological applications.^7–9^

A typical monomolecular G-quadruplex is formed by sequences harboring four G-tracts of three or more consecutive guanosine residues separated by short intervening sequences. Correspondingly, conservative search algorithms are based on a consensus sequence motif d(G_3+_N_1−7_G_3+_N_1−7_G_3+_N_1–7_G_3+_) for predicting putative G4 structures in genomic DNA.^10,11^ However, a growing number of non-consensus sequences has been reported to actually fold into stable G4 species. The availability of their high-resolution structures has shown a variety of unique conformational features distinct from the ‘classical’ G4 architecture. Clearly, a better understanding of principles governing quadruplex folding of such non-standard G-rich sequences will support new algorithms for predicting putative regions within the genome amenable to G4 formation,^12,13^ but may also expand the G4 structural landscape for more effective drug targeting or the engineering of novel G4-based scaffolds.

This review is primarily focusing on the increasing number of G4 structures that do not comply with a consensus sequence motif but rather rely on short G_2_-tracts and/or isolated G nucleotides for their architecture. Various strategies to compensate for G-deficiencies within their G-core or for reduced stacking interactions between tetrads are surveyed to give more insight into relevant contributions to G4 stability. Given the large number of deposited G4 structures with unusual sequence motifs, emphasis is placed on the folding behavior of unmodified sequences, with less attention given to quadruplexes featuring several closely spaced tracts of four or more consecutive guanosines and non-canonical tetrads, *i.e.*, those composed of additional residues other than Gs.

## A short survey on canonical G-quadruplex structures

**Fig. 1:**
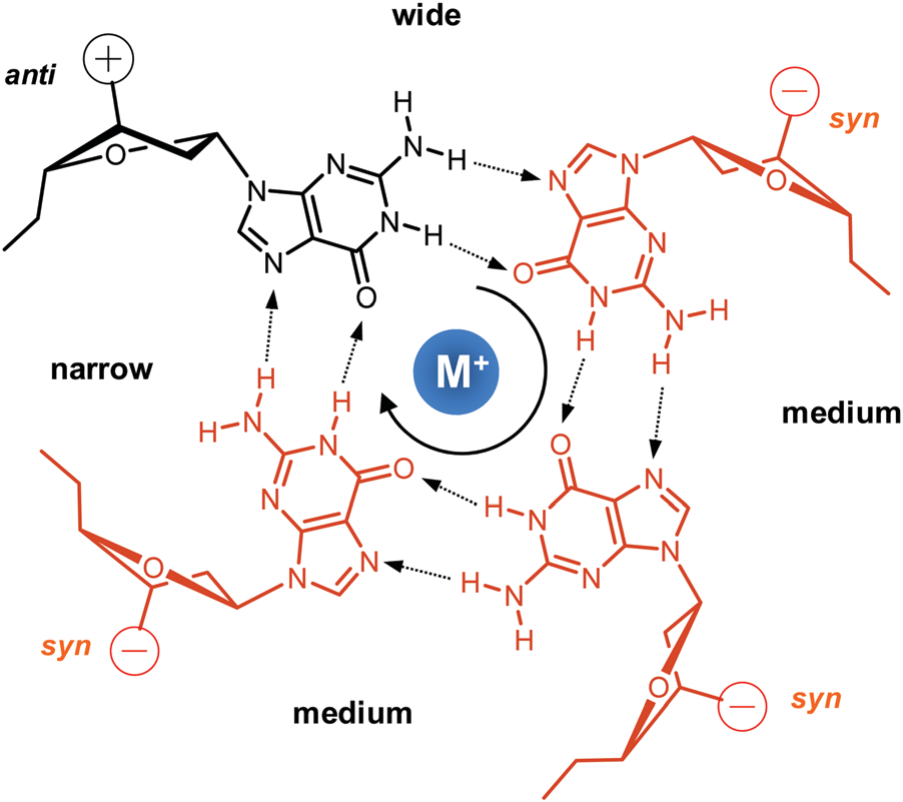
Guanine tetrad with a centrally located metal ion, residues in *syn* or *anti* conformation, and four grooves of narrow, medium, and wide widths; strand polarities are indicated by + and − with the tetrad polarity running in a clockwise direction.

Upon folding of a sequence composed of four closely spaced GGG triplets, guanine bases from the G-tracts will associate to form planar G-quartets (G-tetrads) through a cyclic hydrogen bond pattern involving both their Hoogsteen and Watson–Crick faces (Fig. 1). In most cases, stacking of three G-tetrads gives a three-layered G-core that is additionally stabilized through monovalent cations with a strength of stabilization in the order K^+^ > Na^+^ ≥ NH_4_^+^ > Li^+^.^14^ These are coordinated within the central channel of the G-core that is lined by the G-carbonyl oxygens to create a strong negative potential.

**Fig. 2:**
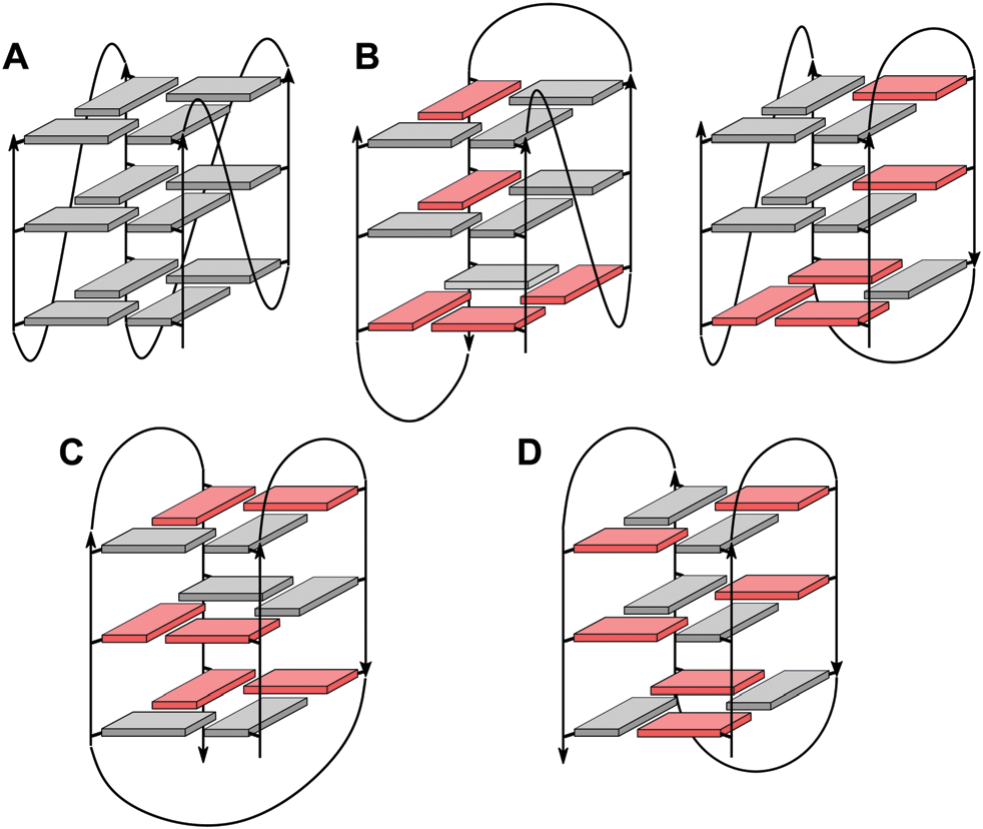
Topologies of canonical three-layered G-quadruplexes. (A) Parallel quadruplex with strands connected by three propeller loops and all-*anti* G-tetrads; (B) (3+1) hybrid quadruplexes with three parallel and one anti-parallel strands connected by one propeller and two lateral loops; (C) basket-type (2+2) anti-parallel quadruplex with each strand adjacent to a parallel and an anti-parallel strand, two lateral and one diagonal loops, and G(*syn*)–G(*syn*)–G(*anti*)–G(*anti*) tetrads; (D) chair-type anti-parallel quadruplex with each strand adjacent to two anti-parallel strands, three lateral loops, and G(*syn*)–G(*anti*)–G(*syn*)–G(*anti*) tetrads; residues in *anti* and *syn* conformation are colored grey and red, respectively.

In case of an intramolecular quadruplex, intervening sequences form loop regions connecting the four G-columns (Fig. 2). A propeller or double-chain-reversal loop links two adjacent G-tracts with parallel orientation whereas lateral (edge-wise) and diagonal loops connect two adjacent or distal anti-parallel G-tracts, respectively. Depending on its topology, a conventional monomolecular quadruplex may be grouped into three major families: a parallel G4 with all four G-tracts being parallel and only containing propeller loops; an anti-parallel G4 with two parallel and two anti-parallel G-runs; and a (3+1) hybrid with three parallel and one anti-parallel G-columns. Because an intramolecular quadruplex is defined by a combination of three different types of loops progressing in either a clockwise or counter-clockwise direction, a large number of topologies is conceivable. For a more systematic nomenclature, a descriptor based on the type of consecutive loops and their progression in relation to a frame of reference has been suggested.^15^ In such a system, the parallel topology with three sequential propeller loops progressing in an anti-clockwise direction can be designated as (–p–p–p) (Fig. 2A). Clearly, several of the theoretical loop combinations are forbidden due to geometrical restrictions. In fact, only 14 of these were predicted to be mechanically feasible but four of those have still not been experimentally verified to-date.^16,17^

Among conformational properties of individual G residues within the quadruplex core, glycosidic torsion angles, *i.e. syn* and *anti* conformers, play a critical role for any quadruplex species due to their importance in G-tetrad formation and their close link with relative strand polarities of the four G-columns. In a parallel quadruplex, all residues within a tetrad must adopt the same glycosidic torsion angle for forming a planar G-quartet arrangement held together by the eight Hoogsteen hydrogen bond interactions. Typically, such G4s are composed of an all-*anti* G-core, although exceptions forming a single all-*syn* quartet exist for modified but also unmodified quadruplexes.^18,19^ On the other hand, residues in anti-parallel G-tracts require different glycosidic conformations when participating in the same G-tetrad. This relationship between relative strand polarities and glycosidic torsion angles has frequently been used as a powerful tool to guide folding of a G-quadruplex through the site-specific incorporation of G analogs favoring either *syn* or *anti* glycosidic torsion angles to enforce a particular topology.

Whereas the pattern of glycosidic angles for residues within a G-tetrad is determined by the orientation of the four G-columns, the sequential glycosidic conformation of consecutive G residues within an individual G-run may vary. It should be noted, however, that changing the glycosidic torsion angle within a column will, as a consequence, also change the tetrad polarity, *i.e.*, the clockwise or anti-clockwise direction of Hoogsteen hydrogen bonds within a tetrad plane when going from hydrogen bond donor to hydrogen bond acceptor. Thus, *anti*–*anti* and *syn*–*syn* steps will result in homopolar tetrad stacking whereas *syn*–*anti* and *anti*–*syn* steps will lead to heteropolar stacking. Computational studies have predicted more favorable interactions for *syn*–*anti* and *anti*–*anti* steps with energetic penalties for *anti*–*syn* and *syn*–*syn* steps, consistent with conformational properties of most reported G-quadruplex structures.^20,21^

In addition to their relationship with relative strand orientation and G-tetrad polarity, glycosidic torsion angles will also affect the width of the four grooves featured by the four-stranded quadruplex. Whereas all grooves in parallel quadruplexes are of medium width, base-paired Gs of different glycosidic conformation as observed in anti-parallel and (3+1) hybrid structures will also form narrow and wide grooves in case of *syn* → *anti* and *anti* → *syn* arrangements within a tetrad, respectively.

From a perspective of intervening sequences, it is their folding into a specific type of loop that defines the topology of the quadruplex architecture. General guidelines have emerged, correlating the length and position of linker sequences to the stability and to favored G4 structures.^22–24^ Due to geometric restraints, formation and stability of particular loops are strongly correlated with the length of the intervening linker sequences. Generally, propeller loops are most stable when composed of only 1–2 residues although even 0 nt propeller loops have been reported in rare cases.^25,26^ Lateral loops often include 2–4 residues depending on bridging a narrow or a wide groove, and diagonal loops require ≥3 residues for linking diagonally positioned G nucleotides. However, even for a conventional sequence, additional complexity may arise due to loop and overhang residues being engaged in subtle tertiary interactions to likewise affect the favored topology. Finally, it is not only the inherent sequence but also the outer conditions like the nature of cations, the ionic strength, and molecular crowding that may significantly impact the topology of a folded quadruplex. Whereas sodium ions have been shown to promote an anti-parallel topology, potassium ions rather tend to destabilize anti-parallel quadruplexes.^14,27^ Folding of the same sequence into either a monomolecular or bimolecular quadruplex may depend on low or high potassium ion concentrations in the buffer solution and is yet another example for an often rather unpredictable folding pathway even when looking at regular G4-forming sequences.^28,29^

Taken together, intense research during the past years has provided a wealth of information regarding the energetics and structural interdependencies in ‘conventional’ G-quadruplexes. Our present knowledge of folding principles enables us to make a guess as for the most stable quadruplex fold of a given G4 consensus sequence and to tackle the rational design of G4 architectures.^30,31^ However, we are still far from reliably predicting topologies based on primary structure due to more subtle additional interactions involving flanking and intervening residues and also to the impact of specific solution conditions.

## Quadruplexes with long loops and quadruplex–duplex hybrids

**Fig. 3:**
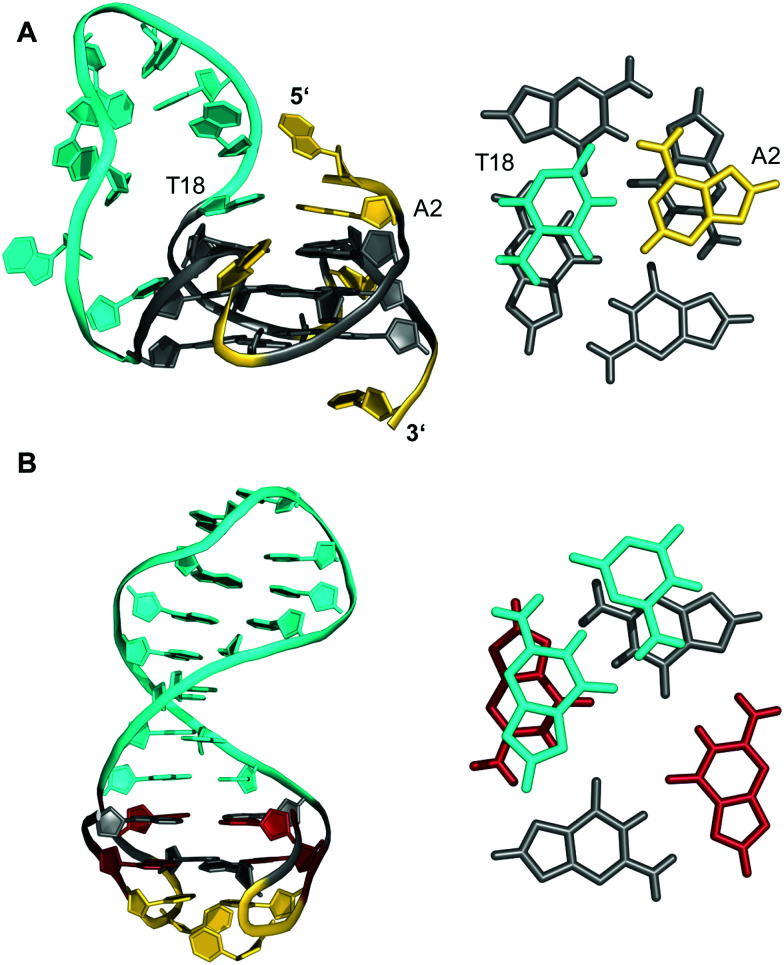
(A) Solution structure of a human *CEB25* minisatellite sequence with a 9 nt propeller loop formed in K^+^ solution (20 mM KP_i_ + 70 mM KCl, pH 7.0; *T*_m_ = 76.5 °C; PDB 2LPW);^35^ an A·T Watson–Crick base pair between a loop and 5′-overhang residue anchors the 3′-terminus of the loop on top of the 5′-outer G-tetrad (right). (B) Solution structure of a quadruplex–duplex hybrid with a two-layered anti-parallel G-quadruplex and a coaxially stacked duplex hairpin bridging the G4 wide groove formed in K^+^ solution (20 mM KP_i_ + 20 mM KCl, pH 7.0; PDB 2M8Z);^38^ the duplex GC base pair stacks onto the G-tetrad at the quadruplex–duplex interface (right); *anti*- and *syn*-guanosines of the G-core as well as loop and flanking residues are colored grey, red, and yellow, respectively; the 9 nt propeller loop in (A) and the stem-loop duplex in (B) are colored cyan.

Longer unstructured loops in G-quadruplex structures tend to be increasingly disfavored because of entropic effects.^32–34^ In fact, only few quadruplexes with long loops of >7 residues, violating the conservative consensus sequence for putative G-quadruplex forming motifs, have been reported to-date. Thus, a G-quadruplex formed by the conserved 26 nt G-rich fragment of the human *CEB25* minisatellite forms a parallel-stranded G-quadruplex with a 9 nt central double-chain-reversal loop (Fig. 3A).^35^ Within this quadruplex, an A·T Watson–Crick and a potential G·A non-canonical base pair between loop and 5′-overhang residues fix the 3′-terminal loop domain above the 5′-outer G-tetrad. Another example involves two parallel-stranded G4 conformers from a *KRAS* promoter sequence, that were found to coexist in equilibrium and feature long third propeller loops composed of eleven and twelve nucleotides, respectively. In this case, high-resolution NMR structures determined from single mutants revealed π–π interactions between some bases of the propeller loop as contributors to the overall stability of the structure.^36^ Also, sequences encompassing five to seven human telomeric (GGGTTA) repeats were shown by NMR to form (3+1) hybrid structures with an up to 21 nt long propeller loop when inner GGG triplets were blocked from participation in G-tetrads through single G → I or G → T substitutions.^37^ Noticeably, such long loops may constitute new recognition motifs, allowing their targeting by a loop-complementary oligonucleotide to form a double-helical loop region.

Contrary to what is expected assuming most stable 1 nt propeller loops,^39^ longer loops of ≥5 residues are rather frequently found to be of a propeller type. Apparently, such loops often allow for stabilizing tertiary interactions with other loop and flanking residues. Following the concept of loop interactions to stabilize longer loop domains, appropriate linker sequences may intrinsically form Watson–Crick paired stem-loop duplexes as part of stable quadruplex–duplex hybrid structures (QDHs). Notably, in contrast to a regular single-stranded linker, quadruplex stabilities of engineered QDHs generally increase with the length of the double-helical hairpin domain.^40^ When forming a lateral-type loop connection, the duplex is favored to bridge a wide groove of the quadruplex G-core to better accommodate distances between the sugar-phosphate backbones of coaxially oriented duplex and quadruplex domains (Fig. 3B).^31,38^ Here, the first base pair at the junction also affects stability due to additional stacking interactions with the quadruplex outer tetrad.^40^ On the other hand, a connecting hairpin element may likewise replace a regular propeller loop, but by connecting G residues at opposite faces of the G-core the first base pair bridging the junction will be invariably disrupted in such an orthogonal arrangement.

Likewise, a duplex-forming diagonal loop with intrinsic Watson–Crick base pairing can be found for a G-rich sequence located in a promoter region of the HIV-1 long terminal repeat (*LTR*).^41^ In the major G-quadruplex conformation *LTR-III*, the 12 nt loop contains a stabilizing duplex hairpin element with three base pairs. However, the longer distance across the distal edges of the quadruplex again prevents residues at the quadruplex–duplex interface to be engaged in a stable base pair.

## Quadruplexes with a two-tetrad G-core

**Fig. 4:**
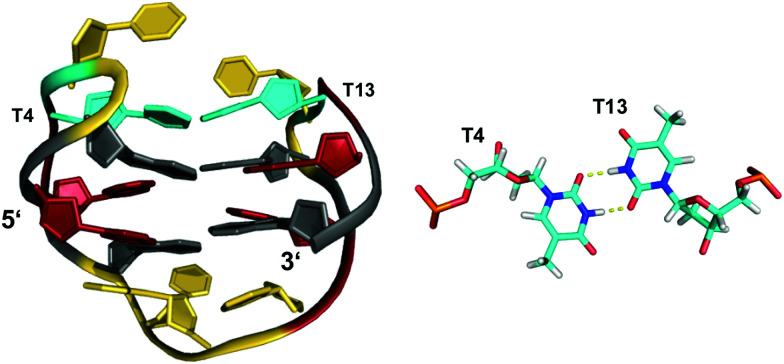
Chair-type anti-parallel G-quadruplex of the TBA aptamer d(GGTTGGTGTGGTTGG) in K^+^ solution (110 mM KCl, pH 6.1) and hydrogen-bonded base pair formed between two T residues from opposite T–T lateral loops (PDB 148D);^46^*anti*- and *syn*-Gs of the quadruplex core, loop residues, and the T·T base pair are colored grey, red, yellow, and cyan, respectively.

The stability of G-quadruplexes is mostly derived from the stacking of its planar tetrads with stacking energies estimated to be ∼80 kJ mol^−1^ per tetrad.^42,43^ Therefore, the stability increases with an increase of stacked tetrads and only a limited number of monomeric two-layered quadruplex architectures has been reported to date. Among these, the thrombin-binding DNA aptamer (TBA) with its four tracts of only two contiguous Gs is one of the most prominent representatives.^44–46^ Each of its four G-tracts has a favorable 5′-*syn*–*anti*-3′ arrangement resulting in opposite hydrogen bond directionalities of its two stacked G(*syn*)–G(*anti*)–G(*syn*)–G(*anti*) tetrads. The G-runs are connected by two T–T lateral loops on one side and a central T–G–T lateral loop on the other side of the anti-parallel chair-type quadruplex (Fig. 4). Additional stabilization comes from the stacking of a TT base pair from the first and third loop on one of the G-quartets. Other stabilizing contributions may also involve some stacking interactions by bases of the central 3 nt lateral loop on the other face of the G-quadruplex core.

Various TBA modifications have been reported in the past, mostly aiming at an improvement of pharmacological properties. Notably, a TBA analog containing a 5′–5' site of polarity inversion in the first lateral loop resulted in a (3+1) hybrid structure by keeping a 5′-*syn*–*anti*-3′ torsion angle progression along all G-runs.^47^ As a consequence, it differs from the unmodified TBA in having one G(*syn*)–G(*syn*)–G(*syn*)–G(*anti*) and one G(*anti*)–G(*anti*)–G(*anti*)–G(*syn*) tetrad alignment with a parallel 5′–3′ strand orientation of the first, second, and fourth strand and a third strand proceeding in the opposite direction. However, stabilizing forces through loop residues are very similar to those found for unmodified TBA.

**Fig. 5:**
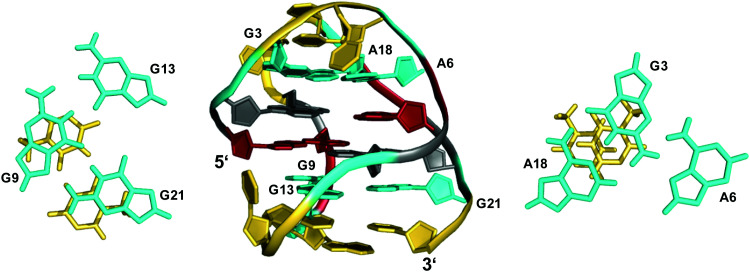
Structure of a human telomeric G-quadruplex (form 3) in K^+^ solution (20 mM KP_i_ + 70 mM KCl, pH 7.0; *T*_m_ = 57.0 °C; PDB 2KF8);^48^ A18·G3·A6 and G21·G9·G13 base triads sandwiched between a G-tetrad and a potential T·T base pair cap the top and bottom of the two-layered G-core; *anti*- and *syn*-residues of the G-quadruplex core, loop and flanking residues, and bases involved in triads are colored grey, red, yellow, and cyan, respectively.

The TBA quadruplex illustrates a frequently observed principle of stabilization through capping structures formed by base pairing alignments of loop and overhang residues. Such interactions may even be favorable enough in two-layered quadruplexes to successfully compete with three-layered G4s in sequences comprising four GGG-tracts. Thus, the unexpected observation of a G-quadruplex with only two tetrad layers for a human telomeric sequence featuring four G_3_-runs emphasizes the potential role of tertiary interactions.^48,49^ Usually, the human telomeric sequence exhibits a (3+1) hybrid form with three stacked G-tetrads in K^+^ solution. However, the 5′-truncated variant d[(GGGTTA)_3_GGGT] was shown to favor a two-layered basket-type structure with all G-columns comprising a 5′-*syn*–*anti*-3′ glycosidic bond arrangement.^48^ The conformation is stabilized by A·G·A and G·G·G triples capping the top and bottom faces of the G-core, respectively (Fig. 5). Moreover, two hydrogen-bonded T residues on top of each triad may add further stacking interactions.

Apparently, extensive base pairing and stacking of loop residues can outweigh stabilities of alternate three-layered G4 structures. It should be mentioned, however, that the telomeric two-G-tetrad conformation has been questioned to be a stable form for the extended human telomeric sequence because the addition of a 5′-flanking residue was shown to mostly abolish formation of a two-layered G4 structure.^49^ Instead, the latter was suggested to likely constitute an intermediate in the interconversion between different telomeric G-quadruplex topologies.

In close analogy to the human telomeric sequence mentioned above, a sequence with single mutation from the RANKL gene d(G_3_TAG_3_AGCG_3_AGAG_3_) adopts a two-layered basket-type topology, again stabilized by a G·G·G and an A·G·A triple on top of the 5′- and 3′-tetrad, respectively.^50^ Here, the critical role of capping base triads and loop residues was uncovered by a structural rearrangement to the anticipated three-layered (3+1) hybrid fold induced by an A5-to-T5 modification. The latter is associated with the destruction of the capping A5·G3·A17 triple, releasing G3 from the A5·G3 base pair. This enables G3 to participate in G-tetrad formation with a concomitant shortening of the 3 nt lateral loop to become a more favorable 2 nt propeller loop.

**Fig. 6:**
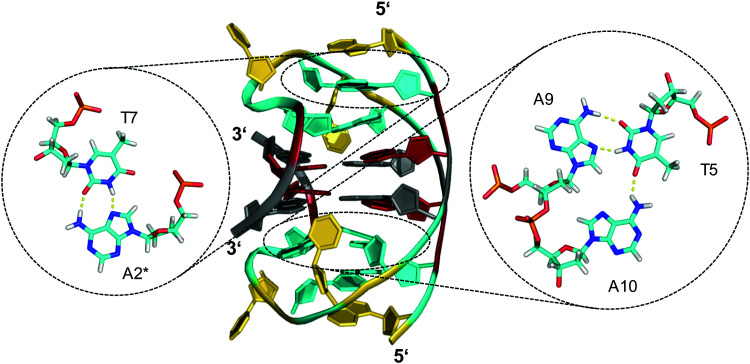
Diamond-shaped bimolecular G-quadruplex with a two-layered G-core formed in Na^+^ solution (5 mM NaP_i_ + 150 mM NaCl, pH 6.9; PDB 1D6D);^53^ each of the tetrads forms a platform that is capped by a T(*anti*)·A(*syn*)·A(*anti*) triad (right) and a reversed Hoogsteen A·T base pair (left); an asterisk denotes a residue from the symmetry-related strand; *anti*- and *syn*-residues of the G-quadruplex core, loop and flanking residues, and bases involved in triads and base pairs are colored grey, red, yellow, and cyan, respectively.

Other examples exist for the stabilization of a two-layered quadruplex core by additional tiers of planar base pairing arrangements from overhang and loop residues.^51–54^ Thus, a truncated form of the *Bombyx mori* telomeric single repeat sequence d(TAGG) was shown to fold into a four-stranded quadruplex with a two-fold symmetry axis consisting of two G(*syn*)–G(*syn*)–G(*anti*)–G(*anti*) tetrads of different tetrad polarity.^51^ The two-layered core is sandwiched between unusual T·A·A triads with one adenosine pairing with the A–T Watson–Crick pair through the minor groove. All three bases and the sugar ring of one adenosine of the triad partially stack over the underlying G residues of the quartet. Inspired by the latter architecture, a sequence d(GGGTTCAGG) was designed and demonstrated to fold into a two-fold symmetric bimolecular G4 structure with heteropolar stacking of two G(*syn*)–G(*anti*)–G(*syn*)–G(*anti*) tetrads capped by a C·G·A triad on each of the two quadruplex faces.^52^ Emphasizing the important role of additional layers made up by triads, the 12mer sequence d(A_2_G_2_T_4_A_2_G_2_) with a pair of AAGG repeats folds into a bimolecular structure with 2-fold symmetry and a core of two G(*syn*)–G(*syn*)–G(*anti*)–G(*anti*) tetrads capped on both sides by A·T·A triads.^53^ The latter, sandwiched between a G-tetrad and an additional outer non-Watson–Crick A–T base pair, contains one adenosine in *syn* conformation that pairs with the thymine through a reverse Hoogsteen alignment (Fig. 6). It should be mentioned that synergistic effects between the unusual base triads and the G4 core result in significant contributions of the stacked triads to the stability of two-layered quadruplexes but also to the promotion of base triad formation through the tetrad platform.

**Fig. 7:**
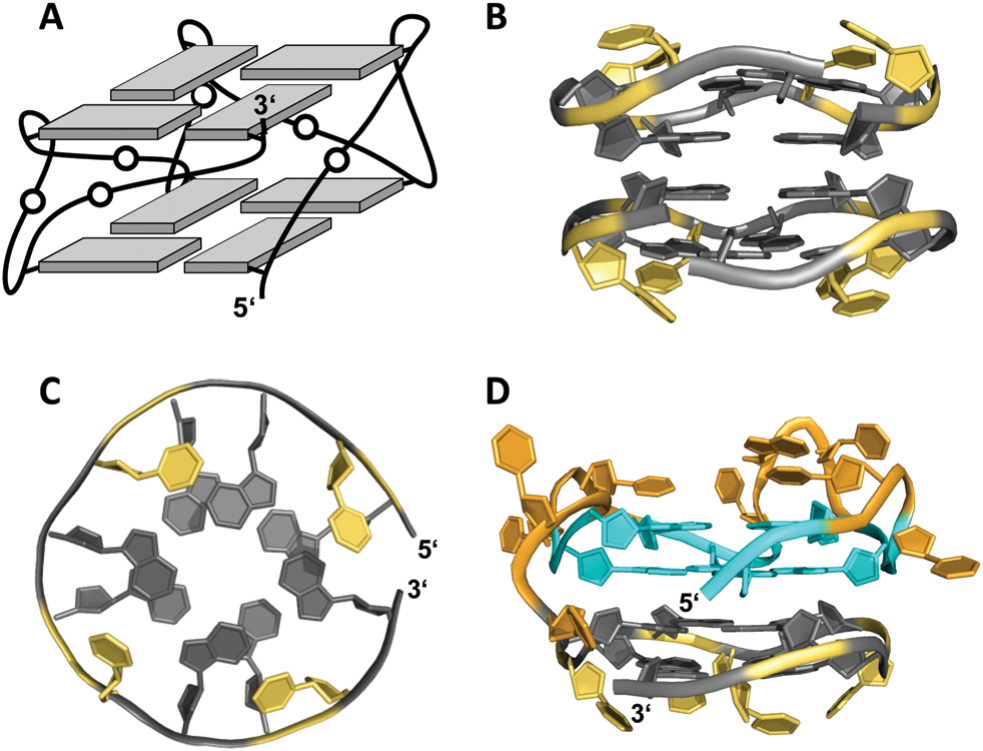
(A) Schematic representation of a minimal left-handed G4. (B) Crystal structure with two stacked left-handed G4 units (crystals grown from 12 mM spermine and 80 mM KCl, pH 7.0; PDB 6FQ2).^55^ (C) Top view with T loop residues oriented towards the outer tetrad of the left-handed domain; a broken G-column is formed by two split Gs at the 5′- and 3′-ends. (D) Hybrid structure with a right-handed TBA subunit connected to the left-handed motif formed in K^+^ solution (20 mM KP_i_ + 70 mM KCl, pH 7.0; PDB 6JCE).^56^ G residues of the quadruplex core and loop residues are colored grey and yellow for the left-handed G4, and cyan and orange for the TBA subunit in (D).

A 12 nt minimal sequence d[GT(GGT)_3_G] derived from the anti-proliferating 28 nt DNA aptamer *AGRO100* forms a unique left-handed parallel G-quadruplex with two G-tetrad layers connected by short loops (Fig. 7A).^55^ Lacking additional capping structures, it dimerizes through 5′–5′ stacking for additional stabilization (Fig. 7B). Likewise, two monomers connected by a linker form a four-layered structure with two stacked left-handed subunits of parallel topology. Single-residue loops are clearly favored for the formation of the left-handed G4. In fact, thymine bases of the 1 nt loops collapse toward the terminal G-tetrad and allow for hydrogen bonds between their O4′ atoms and amino protons of adjacent tetrad guanines (Fig. 7C). Whereas the TBA sequence features four GG doublets, the 12 nt sequence of the left-handed G4 comprises two single Gs at each terminus. By their stacking upon each other they form an unusual split-guanine tract which is assumed to convey the left-handed twist with its fully circling backbone (Fig. 7C).

Apparently, parallel-stranded two-layered quadruplexes from sequences that encompass closely spaced G-doublets have a strong propensity for additional stacking interactions, either through dimerization or in case of longer sequences through a stacked arrangement of two G4 domains made up of their 5′- and 3′-segments. Several examples for the latter derive from the polymorphic *AGRO100* aptamer that is composed of two domains with four G_2_-tracts each. A single G-to-T substitution in the 5′-stretch and addition of thymidine residues at the termini yielded a well-defined sequence that folds into a four-layered G-quadruplex comprising two propeller-type parallel-stranded subunits connected through a central linker.^57^ On the other hand, an alternate G-to-T substitution in the 3′-terminal G-doublet yielded a quadruplex topology termed Z-G4, featuring two stacked G4 domains both with left-handed helicity.^58^ Noticeably, the latter is enforced by the 3′-domain composed of the 12 nt minimal motif mentioned above.^55^

The TBA sequence can also be forced into a parallel topology with its three lateral loops switching into three propeller loops by its linkage to the minimal left-handed G4 sequence. Here, the two G4 units again stack on each other, yet with different helical orientation (Fig. 7D).^56^ Because lateral loops impede stacking, favorable stacking interactions between the two subunits, *i.e.*, the right-handed TBA and the left-handed domain are efficient in driving such refolding into a parallel G4. Also, additional stacking of one base from each propeller loop on the 3′-outer TBA tetrad was observed and may contribute to the stabilization of this TBA topology.

**Fig. 8:**
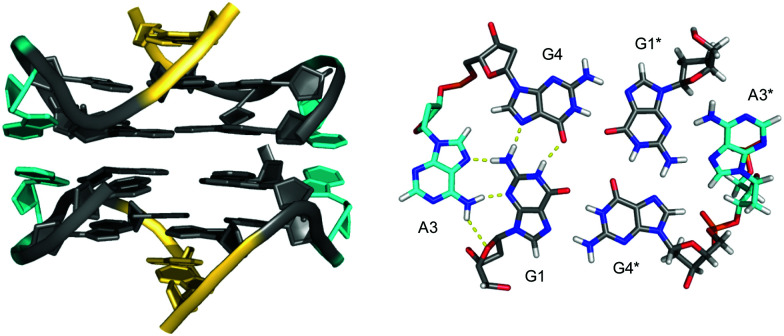
Dimeric G4 structure formed in Na^+^ solution (2 mM NaP_i_ + 150 mM NaCl, pH 6.6) and composed of four symmetry-related strands with stacked hexads at the dimer interface (PDB 1EEG);^59^ guanosines of the all-*anti* quadruplex core, loop and flanking residues, and adenosines involved in hexads are colored grey, yellow and cyan, respectively.

Stabilization can also be provided by bases that are directly linked in-plane to the G-tetrad to form pentads, hexads or heptads. Thus, a dimeric hexad motif with two hexads stacking upon each other was reported for a d(GGAGGAG) sequence in a 150 mM Na^+^ solution.^59^ GGA triplet repeats are abundant in eukaryotic genomes and thought to also be associated with the occurrence of several diseases.^60,61^ In the two tandem GGA triplet repeat sequence, each bimolecular monomer forms a stack of a G·(A)·G·G·(A)·G hexad, a G-tetrad, and an A·A mismatched base pair (Fig. 8). The hexad forms by the in-plane attachment of two adenine bases over their Hoogsteen edge to the G-tetrad through hydrogen bonding with opposite guanine bases. Thus, two out of the four G-tetrad guanines are anchored through a total of six hydrogen bonds. Formation of such hexads is expected to be supported or even driven by extensive π–π stacking interactions between two stacked hexads at the dimer interface.

A corresponding architecture with an intramolecular stack composed of a G·(A)·G·(A)·G·(A)·G heptad and a G-tetrad, additionally stabilized through dimer formation with stacked heptads at the interface, was also found for a four tandem GGA triplet repeat d(GGA)_4_. Likewise, an intramolecularly folded d(GGA)_8_ extended sequence with two subunits composed of stacked tetrad and heptad arranged in a tail-to-tail orientation with inter-heptad stacking.^62,63^ Notably, although octad formation through the G-tetrad alignment of a fourth adenine base either from the 3′-terminus in d(GGA)_4_ or from the adenosine linking the two subunits in d(GGA)_8_ is conceivable, it has not been observed. Apparently, the adenosine requires a subsequent 3′-adjacent G residue as part of the G-tetrad to be anchored within the tetrad plane.

In conclusion, the structure of two-layered quadruplexes as presented above emphasize the need for other stabilizing interactions in addition to the stacking of two tetrads in a G-core composed of favorable *syn*–*anti* or *anti*–*anti* steps along the four GG-columns. Here, loop residues are of particular importance by forming base pairs and/or base triads as additional stacked layers sandwiching the G-quadruplex core. Also, dimerization or inter-subunit stacking is often observed in case of two-layered quadruplexes with a propeller-type parallel topology, enabling unrestricted stacking with interfacial 5′-outer tetrads generally found to be more favorable.^64^ Stacking interactions can be further optimized by expanding G-tetrads with intervening bases to form hexads or heptads, increasing the stacking interface within dimeric structures.

## G-deficient G-quadruplexes and interrupted G-tracts

**Fig. 9:**
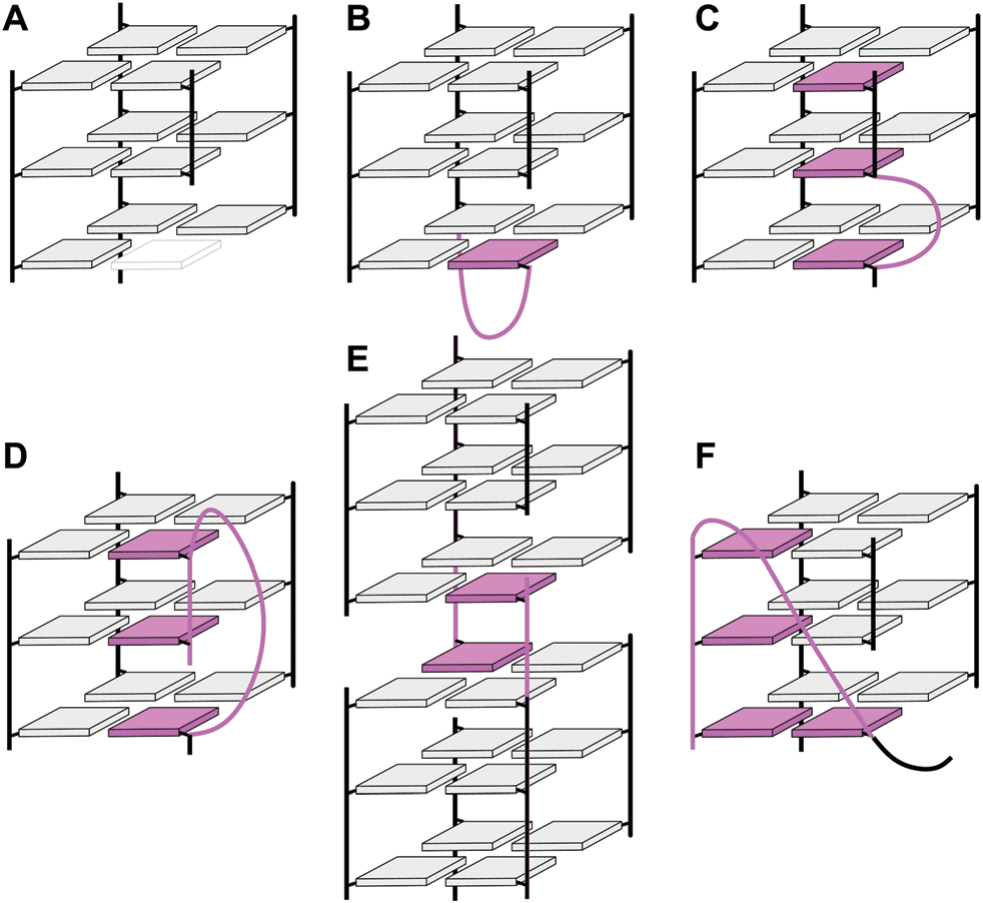
Strategies to fill vacant positions within a quadruplex G-core. (A) Quadruplex with a vacant site, (B) snapback-loop, (C) bulge, (D) D-shaped loop, (E) interlocked G4, (F) V-shaped loop.

In the past, an increasing number of G4-forming sequences harboring a shortened G-tract and thus unable to fold into a canonical three-layered quadruplex with four non-interrupted GGG-columns have been reported. Assuming a thermodynamically controlled G4 folding, the final conformer will maximize favorable interactions, primarily striving to fill vacant G-core positions for increased stacking interactions but also through additional interactions involving intervening and flanking segments. Depending on the primary sequence, there are various possibilities for intramolecular G insertions into unoccupied G-core positions, leading to distinct structural features with bulged or interrupted G-columns. These approaches are schematically depicted in Fig. 9. In the following, corresponding G4 folds are reviewed with particular emphasis on non-modified quadruplexes whose folding pathway is not guided by conformational preferences of incorporated nucleoside analogs (for the latter, see ref. 65).^65^

## G-quadruplexes with a guanine vacancy (vG4)

Deviating from the consensus sequence of a canonical G-quadruplex, sequences with three GGG-tracts and one guanine-deficient GG-tract can assemble into a three-layered quadruplex structure with one tetrad bearing a vacant site. Notably, bioinformatics studies have shown that such sequences are abundant in genomes and may be evolutionarily selected in genes with unique distribution patterns in both eukaryotic and prokaryotic organisms.^66,67^ The vacant site can easily accept a guanine base from guanine-containing metabolites such as GTP or GMP to form an intact and strongly stabilized G-core, demonstrated to effectively alter DNA replication *in vitro* at physiological GTP concentration.^66^ Because G-quadruplexes with guanine vacancies (vG4) are distinct from canonical G4 structures in being able to sense intracellular concentrations of guanine derivates, they have been proposed to play a critical role in gene regulation.

On the other hand, the abundance of vG4 forming sequences in the human genome offers a great potential for therapeutic interventions by more specific, high-affinity targeting. Thus, a bifunctional G4-binding peptide guided through a covalently linked guanine base was shown to feature promising selectivity and affinity toward the G-deficient quadruplex associated with strong suppression of *in vitro* replication.^68^ From an analytical viewpoint, sensors for guanine derivatives based on quadruplexes with a vacant site have been shown to confer exceptional selectivity toward the analyte.^69^

**Fig. 10:**
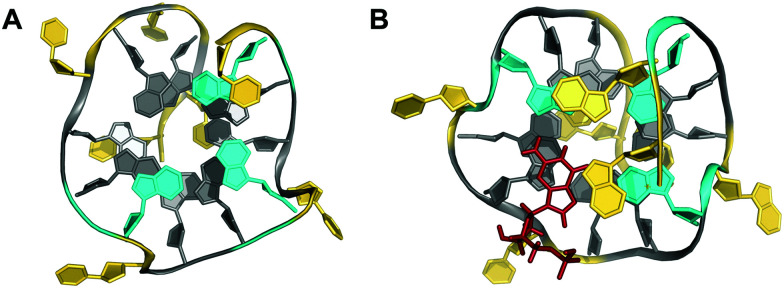
Top view on (A) the vG4 NMR structure formed in K^+^ solution (10 mM KP_i_ + 35 mM KCl, pH 7.0) from the sequence d[TTGTG(TGGG)_3_T] with G-triad (colored cyan) stacked on a G-tetrad (PDB 2N60).^70^ (B) Top view on a dGMP-complexed vG4 structure formed in K^+^ solution (12.5 mM KP_i_ + 37.5 mM KCl, pH 7.0) from the PDGFR-*β* gene promoter sequence (PDB 6V0L);^71^ dGMP (stick model in red) fills the vacant site of the 5′-outer G-layer (colored cyan). Other all-*anti* G-tetrad core residues are colored grey; loop and flanking residues, yellow.

Despite the presence of a destabilizing additional thymine bulge in the short and non-contiguous GG-column at its 5′-end, the sequence d[TTGTG(TGGG)_3_T] containing (12-1) guanines was shown by NMR to fold into a G-deficient intramolecular quadruplex with two G-tetrads and one outer G-triad in a parallel-stranded conformation (Fig. 10A).^70^ In fact, molecular dynamics simulations established the formation of a G-triad-water complex with water molecules occupying the vacant site in the G-triad plane. Again, the vacancy being a G-binding hotspot can be specifically recognized by external guanine bases. High-affinity binding was observed for linear and cyclic d(AG) and cGAMP dinucleotides when targeting a T deletion mutant d[TTGG(TGGG)_3_T] lacking the bulge.^67^

Another example of a structurally characterized G-deficient quadruplex involves a modified human PDGFR-β gene promoter sequence d(AAG_3_AG_3_CG_2_CG_3_ACA) termed Pu19m2.^71^ It was shown to adopt two stable G4 structures formed by the G_2_-tract shifted toward the 5′- or 3′-terminal quadruplex face with a corresponding vacancy in an outer plane adjacent to either the 3′- or 5′-terminus. The triad layer of the vG4 can again be complemented by the selective and strong external binding of physiologically relevant guanine metabolites such as dGMP, GMP, and cGMP but also by guanine-based drugs (Fig. 10B). Interestingly, metabolite binding is able to modulate the equilibrium between the two G_2_-shifted isomers, mostly favoring binding to the G-deficient 5′-triad.

## Snapback loop G-quadruplexes

If the Pu19m2 sequence of the PDGFR-β promoter with its vG4 fold is extended to also include the wild-type 3′-terminus with another G_3_-tract, the resulting sequence d(AAG_3_AG_3_CG_2_CG_3_GCAGGG) designated Pu22m1 was found to adopt a parallel-stranded intramolecular quadruplex with three 1 nt propeller loops and an additional 5 nt lateral loop.^39^ Here, it is a terminal 3′-G in a *syn* conformation that intramolecularly fills the vacant site of the third G_2_-run through a snapback loop structure. Interestingly, the sequence itself features four contiguous runs with ≥3 guanines, expected to fold into a regular three-layered quadruplex without broken strand but with longer second and third loops. Apparently, the high stability of a parallel quadruplex with 1 nt propeller loops outweighs penalties expected for a fourth snapback lateral loop.

A snapback approach in combination with a 5′-terminal hairpin structure was also shown to fill a single vacancy left by a short G_2_-tract. Here, the vacant site acts as an anchor point for the duplex stem-loop in fixing the 5′-terminal G in a *syn* conformation to the tetrad facing the duplex domain.^38^

**Fig. 11:**
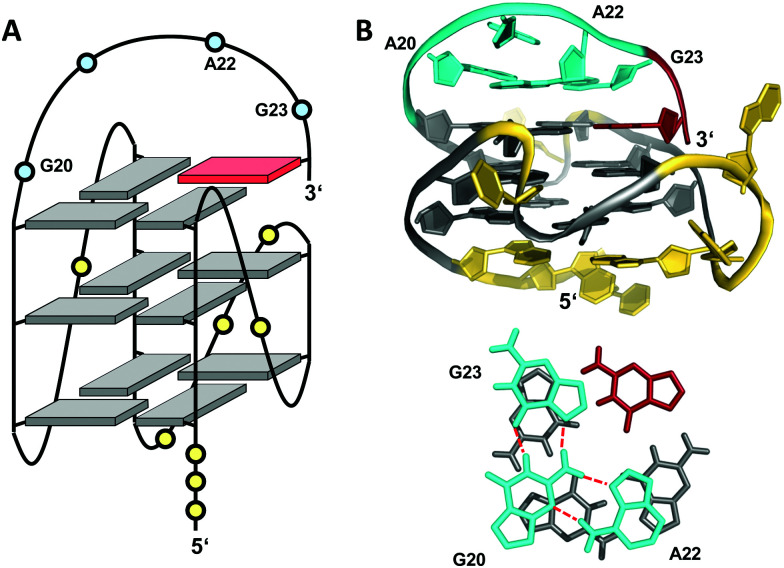
(A) Schematic representation and (B) three-dimensional structure of Pu24I (PDB 2A5P) formed in K^+^ solution (20 mM KP_i_ + 70 mM KCl, pH 7.0) with residues of the diagonal snapback loop forming a G·G·A triad stacked on the 3′-outer tetrad (bottom);^72^*anti*- and *syn*-guanosines of the G-tetrad core are colored grey and red, respectively; loop and flanking residues, yellow; residues forming the snapback loop, cyan.

Snapback loops can also bridge distal corners as exemplified by a *c-myc* promoter sequence d(TGAG_3_TG_4_AG_3_TG_4_AAG_2_) containing five guanine tracts. Although able to fold into a regular parallel G4 with 1 and 2 nt propeller loops, it was shown to favor folding into a parallel-stranded fold-back G-quadruplex with the 3′-terminal guanine base filling an empty guanine position within the 3′-tetrad through a diagonal snapback loop.^72^ The three-dimensional NMR structure of a G10I mutant termed Pu24I demonstrates its parallel fold with 1 nt, 3 nt, and 1 nt propeller loops and a fourth diagonal loop bridging two opposite corners of the 3′-G-tetrad with its terminal *syn*-G complementing the second G-column (Fig. 11). Single base substitutions suggest that a G·G·A triad within the diagonal loop capping the outer G-tetrad seems a critical structural motif for snapback loop formation in Pu24I. Correspondingly, a stacked G·G·A triad from residues of the diagonal snapback loop was likewise found to stabilize one of the two major G4 conformers formed by a G-rich sequence in the *KRAS* nuclease hypersensitive element (NHE) region.^36^ It should be noted, that the addition of further non-G residues at the 3′-terminus may still allow for a fold-back topology but is expected to compromise the thermodynamic stability as suggested by calorimetric studies on mutated 3′-T extended *c-myc* promoter sequences with five guanine tracts.^73^

**Fig. 12:**
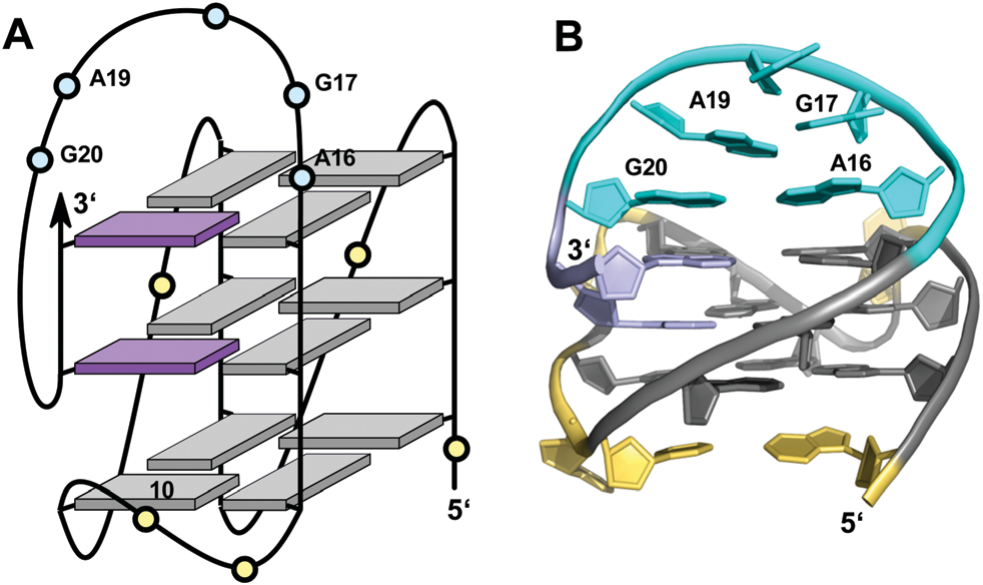
(A) Schematic representation and (B) solution structure of a quadruplex with a distinct type of snapback loop formed by a *c-kit* promoter sequence in K^+^ solution (20 mM KP_i_ + 70 mM KCl, pH 7.0; PDB 2O3M);^74,75^*anti*-guanosines of the G-core and two inserted 3′-terminal Gs are colored grey and lilac, respectively; loop and flanking residues, yellow; residues of the snapback loop forming A16·G20 and G17·A19 base pairs, cyan.

Like the extended PDGFR-β promoter sequence, a G-rich *c-kit* promoter sequence d(AG_3_AG_3_CGCTG_3_AG_2_AG_3_) encompassing four G_3_-tracts and thus capable of forming a regular quadruplex with four continuous G-columns folds into a topology with a snapback loop in K^+^ solution (Fig. 12).^74,75^ Again, the pronounced stability of short propeller-type loops in a parallel topology is suggested to drive folding but base pairing alignments in the loops provide for additional stabilization of this structure with several unique features. Here, isolated G10 is recruited to occupy a single outer G-core position and the corresponding G-column is complemented by insertion of the two 3′-terminal *anti*-G residues aligned in a parallel orientation. The two-residue loop directly following G10 links neighboring corners of the same tetrad. By laterally connecting a broken and continuous G-column of the same strand polarity, it shares features of both propeller and edge-wise loops. The 5 nt snapback connection that follows the fourth G_3_-column to fill the two vacant sites of the third G-tract with parallel-oriented *anti*-G residues shows base pairing alignments. It is unusual in spanning two G-quartets with a 3′-flanking G being part of the central tetrad, allowing unrestricted DNA sequence extensions at the 3′-terminus. Formally, this rather long 5 nt loop may also be viewed as a propeller-type loop progressing against the right-handed helicity of the G-core. The overall topology is also conserved when replacing this loop by a hairpin motif within a closely related *c-kit* based sequence to form a unique quadruplex–duplex junction.^38^ Clearly, it would also be conceivable to fill the two vacant positions through a conventional lateral-type snapback loop with two terminal *syn*-Gs in anti-parallel orientation. However, such a conformer may be disfavored by a less stable *syn-syn* stacking.

## Quadruplexes with a bulge

Non-consecutive guanosines may assemble into G-quadruplexes that feature a G-column of guanines with interdispersed bases protruding outward to form a bulge. Thus, whereas loop residues connect different columns of the G-tetrad core, a bulge connects adjacent guanines along the same column. Bulges have initially been shown in crystal structures of RNA quadruplexes but more recent reports on several three-dimensional structures of bulged G-quadruplexes in solution attest to their potential prevalence and stability under cellular conditions.^36,57,76–83^ Consequently, participation of isolated guanines within interrupted G-tracts in the formation of a stable ‘bulged’ G-tetrad core will significantly expand the number of genomic sequences with a potential for G-quadruplex formation.

**Fig. 13:**
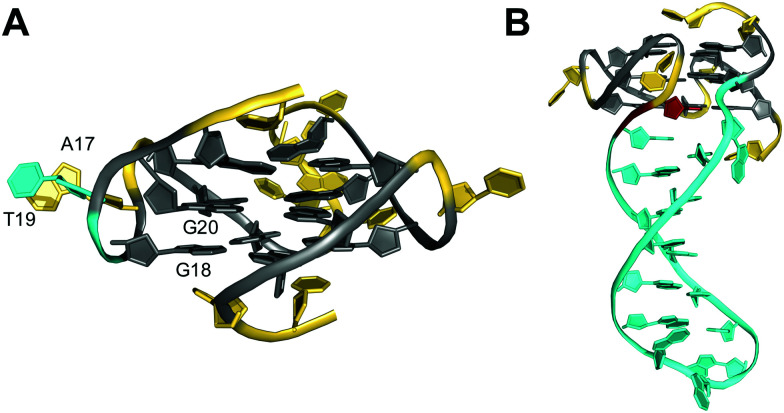
Solution structure of G-quadruplexes with a bulge. (A) Parallel-stranded *LTR-IV* G-quadruplex from the HIV-1 genome formed in K^+^ solution (20 mM KP_i_ + 70 mM KCl, pH 7.0; *T*_m_ = 50.5 °C; PDB 2N4Y);^82^*anti*-G20 following the bulge adopts a north sugar conformation and the bulged T19 stacks onto A17 of the neighboring propeller loop. (B) Parallel-stranded G4 with a bulge forming a stem-loop duplex in K^+^ solution (20 mM KP_i_ + 30 mM KCl, pH 7.0; *T*_m_ = 46.2 °C; PDB 7CLS);^85^ the G residue following the hairpin-forming bulge adopts a *syn* conformation. *anti*- and *syn*-guanosines of the G-tetrad core are colored grey and red, respectively; loop and flanking residues, yellow; residues in bulges, cyan.

The impact of bulges differing in sequence, size, position, or number on G-quadruplex formation was systematically studied by Mukundan and Phan.^80^ Their results suggest that bulges can be located at any position in a G-quadruplex structure. However, the stability of quadruplexes with a bulge decreases with increasing bulge size in analogy to the length dependence of propeller loops. Also, the G4 stability will depend on their location but also on the sequence context and the G-quadruplex topology. In general, bulges are destabilizing, limiting the number of individual bulges *n* compatible with formation of three-layered quadruplexes to *n* ≤ 3. Destabilization can be attributed to a strained backbone but mostly to the unfavorable entropy of solvating the protruding residues. Thus, entropic effects likely determine a stacking interaction of a thymine bulge with a single-nucleotide propeller loop adenine to reduce the hydrophobic surface area in the long terminal repeat sequence *LTR-IV* of the proviral HIV-1 genome (Fig. 13A).^82^ Such rather subtle interactions may in fact explain the different impact of bulges on the thermal stability depending on their position in various topologies.

In general, bulges do hardly perturb the G4 core structure which essentially occupies the same conformational space as found for canonical G-quadruplexes. However, guanosine residues adjacent to bulges have been reported to frequently populate an additional range of backbone torsion angles.^78^ Also, revisiting available quadruplex structures, G-core residues preceding or following the bulge are often found to adopt sugar conformations in the north rather than in the more typical south domain. It should be noted, however, that in most cases no restraints for sugar dihedral angles were employed for generating the NMR solution structures.

A unique 2 nt GA bulge in a G-quadruplex formed by a G-rich sequence in the regulatory region of a RANKL gene connects *anti*- and *syn*-guanosines that occupy G-core positions of a parallel G4.^84^ Consequently, in order to maintain formation and proper stacking of the G-tetrads, the bulge must provide for a turn of the backbone in adopting a pseudo-loop conformation. Remarkably, the corresponding G4-forming sequence encompasses four G_3_-tracts to allow for a regular three-layered quadruplex. Assuming the bulge to be destabilizing, the bulge-containing fold seems to be driven by a shorter overall 1-3-1 when compared to a 1-3-3 propeller loop architecture as expected for a bulge-free parallel topology.

Recently, base-paired duplex bulges of different size were incorporated into various positions of a G-quadruplex scaffold, demonstrating their noticeable stabilization when compared to unstructured bulges.^85^ In fact, thermal stabilities of duplex bulges are slightly increased with increasing bulge sizes, following a similar trend as observed for G4 hairpin loops. The formed quadruplex–duplex junction is reminiscent of an orthogonally aligned propeller-type stem-loop structure with a first disrupted base pair to allow for a progressive transition from the quadruplex to the duplex segment associated with an increase in strand separation (Fig. 13B). However, in contrast to a propeller-type hairpin loop the double-helical foldback bulge continuously stacks onto the 3′-outer G-tetrad and only the first unpaired base projects outward from the groove.

## D-Shaped loop

Unlike bulges that link two split G residues within the same G-column in a consecutive way, another distinct type of loop connects residues of a column located at opposite faces of the G-core. Owing to its characteristic progression it has also been termed D-shaped loop.^26^ This peculiar arrangement positions the 5′- or 3′-terminal G of a d(G_2_N_*x*_G) or d(GN_*x*_G_2_) tract between the other two G residues when forming a G-quadruplex column (see Fig. 9D). In this regard it is reminiscent of a structural motif reported for a short fragment of telomeric DNA from *S. cerevisiae*. Here, an unusual pseudo-circular G-hairpin with a compact core of three GG base pairs is formed and a chain reversal within a continuous G_3_-tract places the 3′-terminal G between the two preceding G residues in the base-paired structure.^86^

**Fig. 14:**
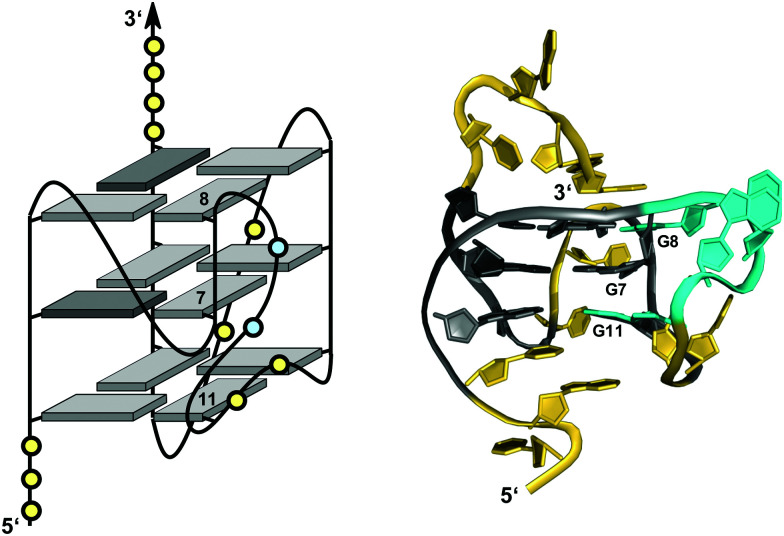
Schematic representation (left) and three-dimensional solution structure (right) of a G-quadruplex with a D-shaped loop derived from a *VEGF* aptamer with locked ^LNA^G residues in K^+^ solution (10 mM KP_i_ + 40 mM KCl, pH 7.0; *T*_m_ = 52 °C; PDB 2M53);^26^*anti*-guanosines and LNA analogs of the G-core are colored in light and dark grey, respectively; loop and flanking residues, yellow; residues of D-shaped loop, cyan.

In a G-quadruplex, such a structural motif was shown for a G-rich VEGF aptamer carrying three locked nucleic acid modifications.^26^ Here, a 2 nt D-shaped loop fills a vacant position within the same column by bridging two corners on opposite sides of the G-core (Fig. 14). Notably, with all three tetrads featuring the same polarity and all core guanosines adopting an *anti* conformation as demonstrated by NMR data analysis, there seems to be no strand inversion between the flanking outer G-core residues as would be expected for this structural motif. Interestingly, however, an easy switch to a *syn* conformation was observed for the 3′-flanking G during structure calculations. A 0 nt propeller loop bridging two tetrad planes precedes and another 2 nt loop directly follows the V-shaped loop. The unusual 2 nt loop ties two parallel-oriented G positions at adjacent corners of the same tetrad in analogy to a corresponding loop in the *c-kit* promoter G-quadruplex.^74,75^

Another example for a D-shaped loop comes from a guanine-rich 36 nt RNA motif named *sc1* capable of quadruplex formation. The solution structure of the *sc1* RNA complexed with an arginine-glycine-rich RGG peptide from the FMRP protein reveals a G-quadruplex domain connected to a flanking duplex stem.^87^ The three-layered all-*anti* G4 is composed of two stacked tetrads of the same polarity and an additional G-tetrad of opposite polarity facing the duplex domain. Here, a strand polarity inversion within one G_3_-tract and two 1 nt D-shaped loops associated with a flipped backbone connect the inverted G-tetrad with the other two G-tetrad layers.

## Interlocked structures

**Fig. 15:**
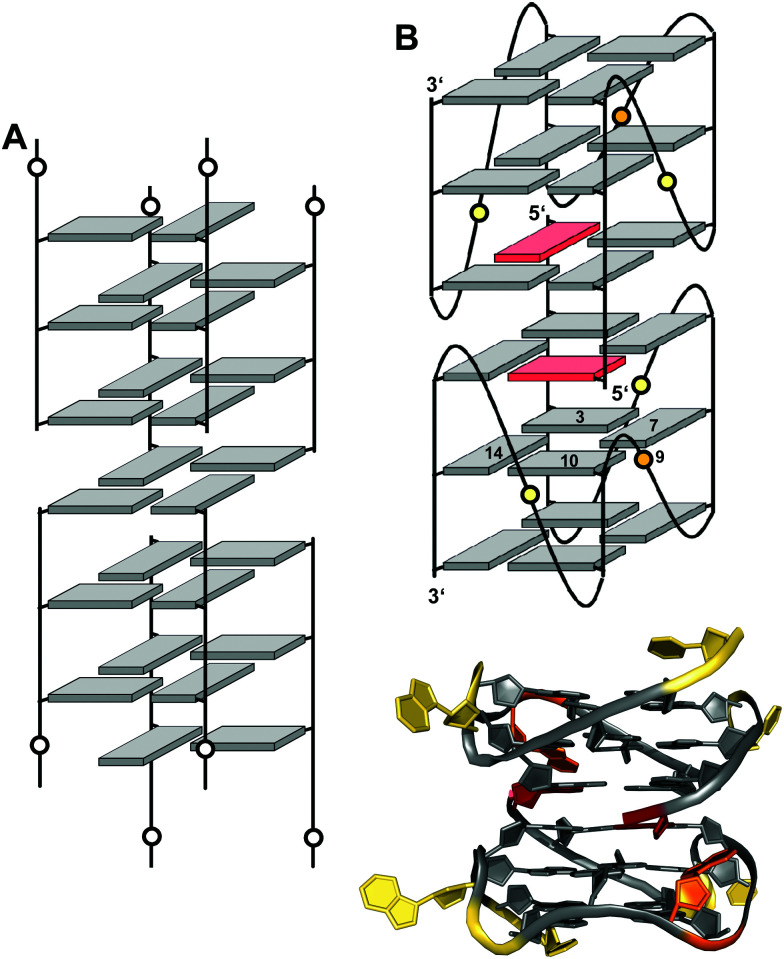
Interlocked G-quadruplexes. (A) Schematic representation of two interlocked d(GGGT)_4_ out-of-register quadruplexes forming an octameric species.^88^ (B) Schematic representation (top) and three-dimensional structure (bottom) of an interlocked quadruplex formed in K^+^ solution (90 mM KCl, pH 7.0) by an HIV-integrase aptamer (PDB 1Y8D);^98^ the pair of G-tetrads at the dimer interface are mutually filled with a 5′-terminal *syn*-G1 from the other strand; *anti*- and *syn*-guanosines of the G-tetrad core are colored grey and red, respectively; loop and flanking residues, yellow; loop adenines A9 aligned in-plane of the tetrad to form a pentad, orange.

Interlocked structures are composed of more than one separate G-rich strand and in the past have often been associated with the formation of G-wires. The latter can form if G-rich strands associate out-of-register to present ‘sticky’ ends. Two such slipped structures may subsequently dimerize through their terminal free G residues to form an extra G-tetrad. Thus, d(GGGT) may align into an octameric complex with five stacked G-tetrads in addition to the tetramolecular d(GGGT)_4_ with in-register strand association (Fig. 15A).^88^ If association is enabled at both 5′- and 3′-ends, self-assembly can lead to large nanostructures by the growth of an interlocked G4 in both directions.

A first model of a G-wire formed by the telomeric DNA oligonucleotide d(G_4_T_2_G_4_) was proposed more than 25 years ago^89,90^ but its structural diversity could only be demonstrated by atomic force microscopy in the recent past.^91^ Expanding on the self-associative potential of G-rich sequences in a slipped alignment, oligomerization was also shown to be supported by GC overhangs that serve as cohesive ‘sticky’ ends to form two interfacial GCGC-tetrads by hydrogen bonding through Watson–Crick and Hoogsteen guanine edges.^92–94^ In G-wires, self-recognition and self-assembly relies on G-quartet formation and is expected to be promoted by hydrophobic effects but also by the enthalpic gain of multiple G-tetrad stacking. Correspondingly, these interlocked structures show high thermal stabilities but due to their slow kinetics of formation their population and length strongly depends on concentration, temperature, and cations present. Of note, some of the higher-order G4 structures suggested to coexist in particular with parallel-stranded G-quadruplexes^23,95,96^ may possibly also be traced to the formation of such high-melting interlocked structures.

Narrowing the definition of interlocked G-quadruplexes, G-tetrads at the interface between two G-deficient quadruplex folds may be mutually completed by Gs from the other subunit. This enables the sequence d(G_3_AG_2_T_3_G_3_AT), bearing only three G-tracts, to fold into a dimeric four-layered quadruplex.^97^ Each monomer adopts a compact domain with a 1 nt propeller loop, a 3 nt lateral loop, and a 0 nt V-shaped loop (see below). The dimeric interface features a pair of stacked A·(G·G·G·G) pentads through the interaction and in-plane alignment of the propeller loop adenine with a G-quartet. Also, each pentad is completed through the insertion of a 5′-terminal *syn*-G residue of the other monomer. The pentad stacks upon a tetrad in each monomer supported by one broken and three continuous GG-columns.

Likewise, the *93del* aptamer d(GGGGTGGGAGGAGGGT), an HIV-1 integrase inhibitor, forms a very stable six-layered G-quadruplex interlocked dimer in K^+^ solution (Fig. 15B).^98^ Each monomeric subunit contains one A·(G·G·G·G) pentad sandwiched between two G-tetrads with the G-tetrad at the interlocking interface complemented by the 5′-terminal *syn*-G from the first G_4_-tract of the other monomer. All G-columns within each monomer are parallel and linked by two 1 nt propeller loops bridging three G-tetrad layers. A second adenosine propeller loop that bridges two G-tetrad layers participates in A·(G·G·G·G) pentad formation.

Based on the *93del* aptamer, sequence variants forming corresponding interlocked G-quadruplex dimers were rationally designed. These encompass a first long G_4_-tract to compensate with its 5′-G for a G-deficient tetrad of the other monomer, two medium G_3_-tracts, and another short G_2_-tract being positioned as second, third, or fourth G-run.^99^ Indeed, very stable interlocked quadruplexes were demonstrated to form and may constitute robust scaffolds for technological applications.

Expanding on the architecture of locked quadruplexes, a unique intra-locked G4 structure was recently reported for the 28mer G-rich sequence d[(TGG)_4_TTG(TGG)_3_TTGT] harboring multiple G_2_-tracts and two single G residues.^100^ This sequence was shown to fold into a structure with two stacked bi-layered subunits formed by its 5′- and 3′-domains. Additionally, intramolecular locking is achieved by the incorporation of a guanine from the 5′-subunit into the G-deficient interfacial G-layer of the 3′-subunit.

## V-loop quadruplexes

V-loops are one of the most prominent non-canonical structural elements in G-quadruplexes and have received growing attention in recent years. V-shaped loops connect two adjacent G-columns by bridging two or three G-tetrad layers (see Fig. 9F). However, in contrast to a propeller-type connection one of the G-columns is broken and the two linked G-tracts are generally oriented anti-parallel to each other. The formation of 0 nt V-shaped loops is most common, but in some cases 1 nt or 2 nt V-loops have also been reported.^54,100,101^

**Fig. 16:**
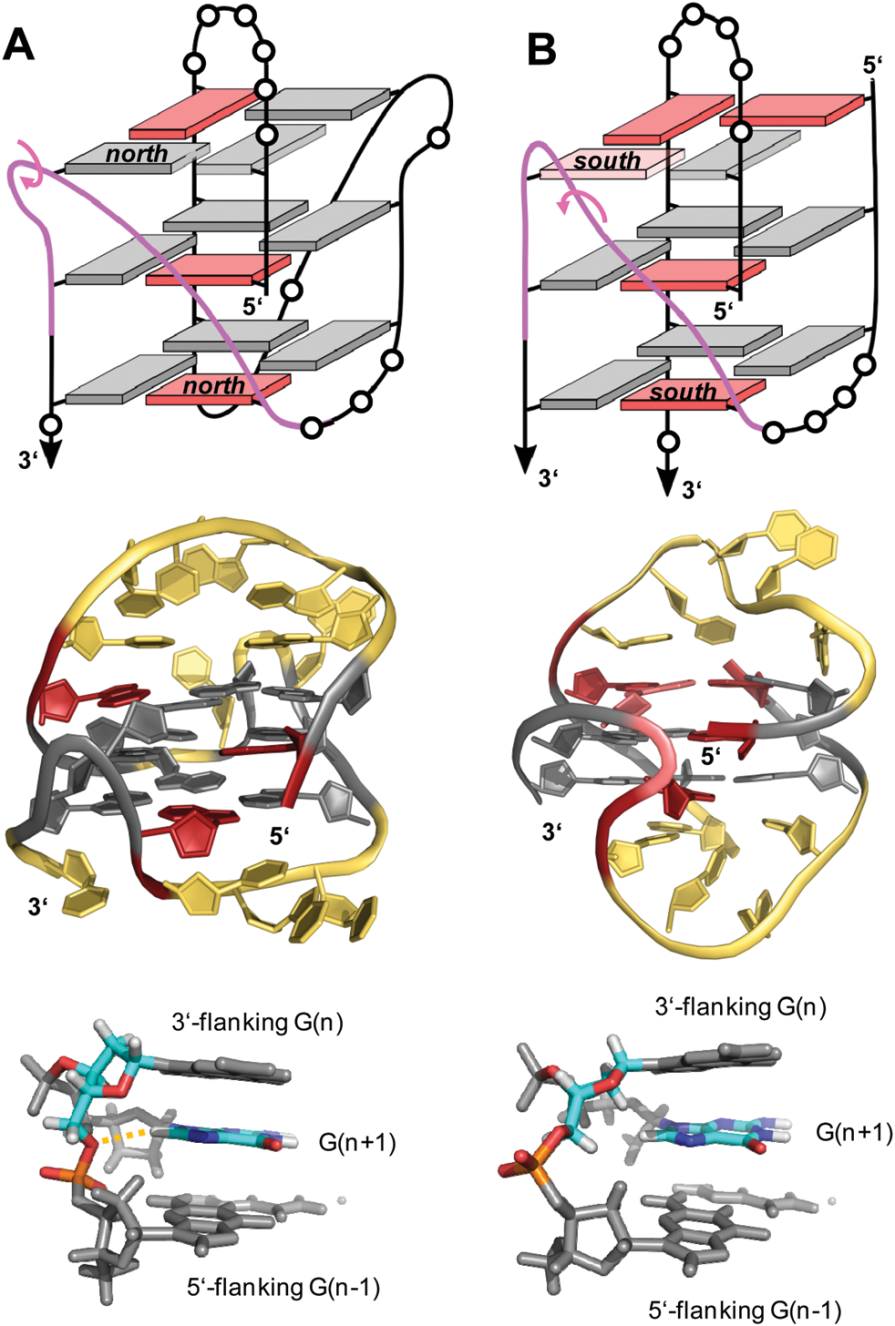
V-loop topology with polarity inversion sites indicated by circular arrows (top), solution structure (center), and backbone conformation for the V-loop (bottom) comprising a 3 nt stretch that is colored magenta in the topological representations on top. (A) Monomolecular G4 formed in K^+^ solution (20 mM KP_i_ + 80 mM KCl, pH 6.8; *T*_m_ = 54.5 °C) with conventional V-loop and O5′(*n*)-H8(*n* + 1) interactions (PDB 5ZEV).^106^ (B) Bimolecular G4 in K^+^ solution (15 mM KCl, pH 5.5) with alternative V-loop and solvent-exposed phosphate (PDB 1U64).^109^ G-core guanosines in *anti*, *syn*, and ‘low-*syn*’ conformation are colored grey, red, and light red, respectively.

V-loops can exhibit high intrinsic stability and may even compete with canonical topologies. Thus, modifying all matching *anti*-G positions with strongly *anti*-favoring ^LNA^G analogs in the telomeric sequence d(G_4_T_4_G_4_) from *Oxytricha nova* resulted in a rearrangement of the bimolecular anti-parallel quadruplex into a unique scaffold with a topology termed V4 fold.^102^ Here, all four G-stretches within two strands fold back in a V-shaped loop with an LNA residue at their 3′-end and interact with each of the other three G-stretches through the formation of four G-tetrads. The V-loop 5′-anchoring position is generally occupied by a *syn*-G being part of a discontinuous G-column. In order to trace favorable and unfavorable contributions to V-loop formation, various sugar-modified G analogs have recently been introduced at specific positions of a (3+1) hybrid quadruplex, triggering rearrangements into a V-loop structure.^103–105^ Detailed analysis of dual-modified V-loop quadruplexes bearing different combinations of G-analogs demonstrated that often overlooked sugar conformational preferences rather than glycosidic conformations were major contributors to V-loop stability. Thus, a stable V-loop structure was even formed when inserting ^LNA^G with its fixed C3′-endo conformation (*north*) at the V-loop 5′-anchoring site to enforce a strongly disfavored *syn* conformation when followed by another 3′-flanking *anti*-^LNA^G.^105^ On the other hand, a sugar pucker in the *north* domain for both 5′- and 3′-flanking residues seems to match backbone conformational requirements of a conventional 0 nt V-shaped loop (Fig. 16A, top). In fact, a corresponding 5′-(*syn*,*north*)-(*anti*,*north*)-3′ conformation for V-loop flanking residues is likewise found for other V-shaped loops in unmodified quadruplexes and represents a characteristic feature for such conventional loops.^106–108^ Owing to the *syn* and *anti* anchor residues participating in G-tetrads of reversed polarity, there is no apparent strand polarity inversion inherent to the V-loop but rather between the 3′-flanking G and the following G within the same G-tract. Interestingly, a sharp turn of the sugar-phosphate backbone at the inversion site and a *north*-type sugar pucker of the 3′-anchoring residue places its O4′ and O5′ oxygen atoms in close vicinity to H8 of the following G to also allow for corresponding C–H⋯O interactions (Fig. 16A, bottom).

A second type of V-loop conformation has originally been suggested based on a sequence bearing a *south/south-east*-favoring 2′-fluoro-arabinoguanosine analog at the 3′-flanking position.^104,105^ In these alternative V-loop conformers, *south*-puckered residues are mostly found for both V-loop framing positions, allowing to differentiate V-shaped loops according to two distinct sugar conformational preferences (Fig. 16B, top). Whereas a *syn* conformation at the 5′-anchor site seems mandatory for all regular V-loops, glycosidic torsion angles at the 3′-end of the alternative V-loop are typically outside the *anti*/*high-anti* range and rather adopt torsion angles in a less defined ‘low-*syn*’ range.^41,109,110^ As a consequence, a sugar-phosphate backbone inversion can formally be localized within the V-loop in this case. Also, larger inter-atomic distances with a more solvent-exposed phosphate of the 3′-flanking residue prevent C–H⋯O pseudo-hydrogen bond contacts between the latter and the subsequent G as observed for a conventional type of V-loop, with possible implications for other intermolecular interactions (Fig. 16B, bottom).

Due to a lessening of conformational restraints exerted by the V-shaped loop, few quadruplexes with a less compact architecture, *e.g.*, with more flexible 1 nt or 2 nt V-loops or with neighboring bulges adjacent to the loop, may feature conformers located slightly outside of either of the two characteristic conformational clusters.^100,101^ Of note, foldback bulges in two recently reported parallel-stranded quadruplexes enforce a single *syn* conformer for the 3′-linked G of a central discontinuous G-tract (see above and Fig. 13B).^84,85^ As a result, the loop following this G-core residue with its inverted backbone orientation may be viewed as a 1 nt V-shaped loop rather than a regular propeller-type loop.

**Fig. 17:**
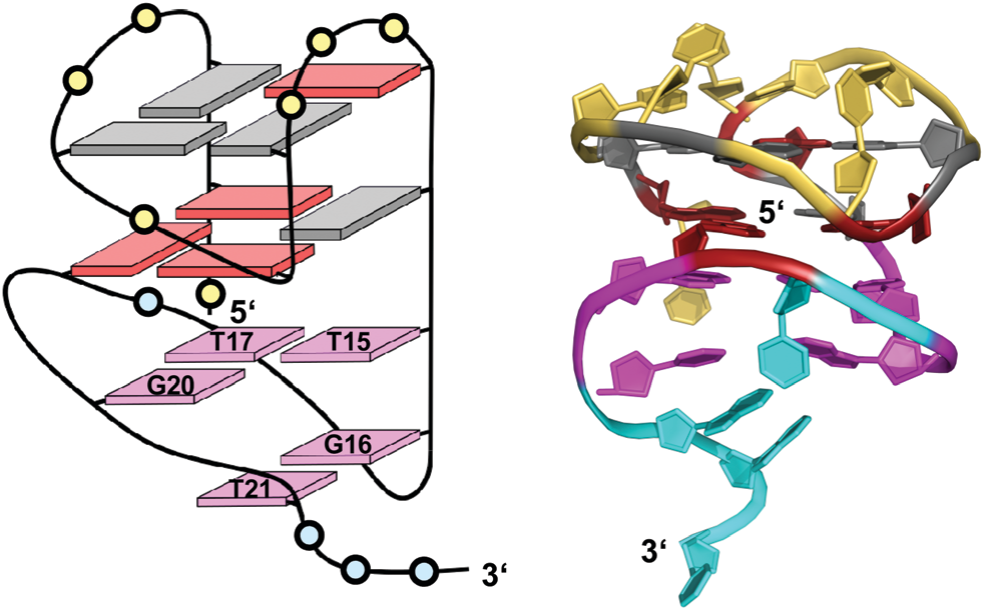
Schematic representation and three-dimensional solution structure for a V-loop quadruplex of the *AGRO100* derived sequence d(TG_2_TTGTG_2_TTTG_2_TGTTG_2_TG_2_T) in K^+^ solution (20 mM KP_i_ + 70 mM KCl, pH 7.0; *T*_m_ = 39 °C; PDB 6JCD);^54^*anti*- and *syn*-guanosines of the G-tetrad core are colored grey and red, respectively; loop and flanking residues of the G4 core structure and of the 3′-peripheral motif are colored yellow and cyan, respectively; T·T·G triad and G·T base pair, magenta.

Finally, a unique two-layered anti-parallel quadruplex derived from the *AGRO100* aptamer combines a 1 nt V-shaped loop with a 3′-terminal domain forming characteristic base pairing alignments.^54^ The 3′-peripheral motif progresses along two sharp U-turns to form two additional layers composed of a T·T·G triad and a G·T base pair capping the 5′-outer tetrad (Fig. 17). It is attached by a non-terminal guanosine to the G4 core, filling a vacant G4 position in a snapback-type arrangement. The compact structural domain of the peripheral sequence was shown to possibly serve as a modular unit, able to replace a diagonal snapback loop in other G4 structures. Notably, in contrast to most V-loop structures the unusual 1 nt V-loop spanning two tetrad planes features a 5′-anchoring guanosine in *anti* conformation.

## Summary and perspectives

G-rich sequences covering a wide range of G-runs of different number and length have been demonstrated to fold into four-stranded G-quadruplexes. As surveyed in this review, G-deficiencies as a result of short G-tracts may be compensated by unique structural motifs supplementing vacant sites. These include V- and D-shaped loops, snapback loops, bulges, and interlocked species. On the other hand, additional stacking interactions through G4 association but also through capping base triads or base pairs may considerably stabilize a particular G4 architecture. Formation of corresponding motifs will depend on the particular sequence context and on the superposition of additional interactions that are often difficult to anticipate based on the primary sequence. However, these interactions may even be strong enough to induce folding into a non-conventional structure even if the sequence allowed folding into a canonical topology.

Based on the rapidly growing number of reported crystallographic and NMR G-quadruplex structures, our knowledge on the structural organization and stabilizing inherent interactions of unusual structural motifs has considerably grown. Thus, we are beginning to recognize and understand major contributors to particular topological features. These include tertiary interactions between different domains of the folded quadruplex to form triads, base pairs, or non-canonical tetrads but also more subtle conformational preferences of individual residues with their often decisive impact on equilibria between G4 conformers being close in energy. Sequences that fold into non-canonical G4 structures featuring interrupted G-columns have already been successfully designed.^38,99,111^ However, whereas our ability to decipher the code that relates a G-rich oligonucleotide sequence with four closely spaced G_3_-tracts to a preferred G4 topology increases, understanding and predicting the folding of irregular G-rich sequences still poses a challenge.

Detailed insight into folding pathways and into interactions enforcing particular structural motifs will be important for the identification of G-rich fragments prone to G-quadruplex formation and also for a successful engineering of quadruplex architectures for various technology-based G4 applications. On the other hand, non-canonical G4 structures offer additional opportunities in their specific targeting for both pharmaceutical and technological purposes. The majority of known G4 ligands binds through stacking interactions onto a G-tetrad. Also, attempts to increase selectivity with less off-target effects based on different groove dimensions or loop conformations has only brought limited success to-date. Exploiting various non-conventional structural motifs may in fact expand our toolbox for achieving more selectivity. G-deficient quadruplexes with a vacant site constitute promising targets for a specific and high-affinity binding of bifunctional ligands that are guided by a covalently linked guanine base. Also, interrupted G-tracts with their opening may potentially support insertion of planar ligands between tetrad planes of the quadruplex. Finally, quadruplex–duplex junctions are expected to be widespread in a cellular environment either through hairpin-type loop domains within the G4 architecture or through G-quadruplexes extruding from a B-type duplex as anticipated for oncogenic promoter sequences. Such interfaces between different structural domains have attracted growing interest in recent years and may provide for unique target sites for G4 drugs.^112–114^ In fact, binding to quadruplex–duplex junctions by appropriate ligands has already been shown to be guided by strong interactions, making junctions one of several promising target sites for the future design of more efficient G4-binding drugs.

## Conflicts of interest

There are no conflicts to declare.

## Supplementary Material

## References

[cit1] Lam E. Y. N., Beraldi D., Tannahill D., Balasubramanian S. (2013). Nat. Commun..

[cit2] Laguerre A., Hukezalie K., Winckler P., Katranji F., Chanteloup G., Pirrotta M., Perrier-Cornet J.-M., Wong J. M. Y., Monchaud D. (2015). J. Am. Chem. Soc..

[cit3] Hänsel-Hertsch R., Antonio M. D., Balasubramanian S. (2017). Nat. Rev. Mol. Cell Biol..

[cit4] Neidle S. (2017). Nat. Rev. Chem..

[cit5] Russo Krauss I., Merlino A., Giancola C., Randazzo A., Mazzarella L., Sica F. (2011). Nucleic Acids Res..

[cit6] de Soultrait V. R., Lozach P.-Y., Altmeyer R., Tarrago-Litvak L., Litvak S., Andréola M. L. (2002). J. Mol. Biol..

[cit7] Chiorcea-Paquim A.-M., Eritja R., Oliveira-Brett A. M. (2018). J. Nucleic Acids.

[cit8] Neo J. L., Kamaladasan K., Uttamchandani M. (2012). Curr. Pharm. Des..

[cit9] Borovok N., Iram N., Zikich D., Ghabboun J., Livshits G. I., Porath D., Kotlyar A. B. (2008). Nucleic Acids Res..

[cit10] Huppert J. L., Balasubramanian S. (2005). Nucleic Acids Res..

[cit11] Todd A. K., Johnston M., Neidle S. (2005). Nucleic Acids Res..

[cit12] Sahakyan A. B., Chambers V. S., Marsico G., Santner T., Antonio M. D., Balasubramanian S. (2017). Sci. Rep..

[cit13] Puig Lombardi E., Londoño-Vallejo A. (2020). Nucleic Acids Res..

[cit14] Bhattacharyya D., Arachchilage G. M., Basu S. (2016). Front. Chem..

[cit15] Webba da Silva M. (2007). Chem. – Eur. J.

[cit16] Fogolari F., Haridas H., Corazza A., Viglino P., Corà D., Caselle M., Esposito G., Xodo L. E. (2009). BMC Struct. Biol..

[cit17] Dvorkin S. A., Karsisiotis A. I., Webba da Silva M. (2018). Sci. Adv..

[cit18] Karg B., Haase L., Funke A., Dickerhoff J., Weisz K. (2016). Biochemistry.

[cit19] Tong X., Lan W., Zhang X., Wu H., Liu M., Cao C. (2011). Nucleic Acids Res..

[cit20] Cang X., Šponer J., Cheatham T. E. (2011). Nucleic Acids Res..

[cit21] Šponer J., Mládek A., Špačková N., Cang X., Cheatham T. E., Grimme S. (2013). J. Am. Chem. Soc..

[cit22] Hazel P., Huppert J., Balasubramanian S., Neidle S. (2004). J. Am. Chem. Soc..

[cit23] Smargiasso N., Rosu F., Hsia W., Colson P., Baker E. S., Bowers M. T., De Pauw E., Gabelica V. (2008). J. Am. Chem. Soc..

[cit24] Cheng M., Cheng Y., Hao J., Jia G., Zhou J., Mergny J. L., Li C. (2018). Nucleic Acids Res..

[cit25] Piazza A., Cui X., Adrian M., Samazan F., Heddi B., Phan A. T., Nicolas A. G. (2017). eLife.

[cit26] Marušič M., Veedu R. N., Wengel J., Plavec J. (2013). Nucleic Acids Res..

[cit27] Fujii T., Podbevšek P., Plavec J., Sugimoto N. (2017). J. Inorg. Biochem..

[cit28] Kuryavyi V., Phan A. T., Patel D. J. (2010). Nucleic Acids Res..

[cit29] Trajkovski M., Webba da Silva M., Plavec J. (2012). J. Am. Chem. Soc..

[cit30] Webba da Silva M., Trajkovski M., Sannohe Y., Ma’ani Hessari N., Sugiyama H., Plavec J. (2009). Angew. Chem., Int. Ed..

[cit31] Karg B., Mohr S., Weisz K. (2019). Angew. Chem., Int. Ed..

[cit32] Bugaut A., Balasubramanian S. (2008). Biochemistry.

[cit33] Hazel P., Huppert J., Balasubramanian S., Neidle S. (2004). J. Am. Chem. Soc..

[cit34] Guédin A., Gros J., Alberti P., Mergny J. L. (2010). Nucleic Acids Res..

[cit35] Amrane S., Adrian M., Heddi B., Serero A., Nicolas A., Mergny J. L., Phan A. T. (2012). J. Am. Chem. Soc..

[cit36] Marquevielle J., Robert C., Lagrabette O., Wahid M., Bourdoncle A., Xodo L. E., Mergny J.-L., Salgado G. F. (2020). Nucleic Acids Res..

[cit37] Yue D. J. E., Lim K. W., Phan A. T. (2011). J. Am. Chem. Soc..

[cit38] Lim K. W., Phan A. T. (2013). Angew. Chem., Int. Ed..

[cit39] Chen Y., Agrawal P., Brown R. V., Hatzakis E., Hurley L., Yang D. (2012). J. Am. Chem. Soc..

[cit40] Lim K. W., Khong Z. J., Phan A. T. (2014). Biochemistry.

[cit41] Butovskaya E., Heddi B., Bakalar B., Richter S. N., Phan A. T. (2018). J. Am. Chem. Soc..

[cit42] Jin R., Gaffney B. L., Wang C., Jones R. A., Breslauer K. J. (1992). Proc. Natl. Acad. Sci. U. S. A..

[cit43] Petraccone L., Erra E., Esposito V., Randazzo A., Mayol L., Nasti L., Barone G., Giancola C. (2004). Biochemistry.

[cit44] Macaya R. F., Schultze P., Smith F. W., Roe J. A., Feigon J. (1993). Proc. Natl. Acad. Sci. U. S. A..

[cit45] Smirnov I., Shafer R. H. (2000). Biochemistry.

[cit46] Schultze P., Macaya R. F., Feigon J. (1994). J. Mol. Biol..

[cit47] Martino L., Virno A., Randazzo A., Virgilio A., Esposito V., Giancola C., Bucci M., Cirino G., Mayol L. (2006). Nucleic Acids Res..

[cit48] Lim K. W., Amrane S., Bouaziz S., Xu W., Mu Y., Patel D. J., Luu K. N., Phan A. T. (2009). J. Am. Chem. Soc..

[cit49] Zhang Z., Dai J., Veliath E., Jones R. A., Yang D. (2009). Nucleic Acids Res..

[cit50] Lenarčič Živković M., Rozman J., Plavec J. (2018). Angew. Chem., Int. Ed..

[cit51] Kettani A., Bouaziz S., Wang W., Jones R. A., Patel D. J. (1997). Nat. Struct. Biol..

[cit52] Kettani A., Basu G., Gorin A., Majumdar A., Skripkin E., Patel D. J. (2000). J. Mol. Biol..

[cit53] Kuryavyi V., Kettani A., Wang W., Jones R., Patel D. J. (2000). J. Mol. Biol..

[cit54] Truong T. H. A., Winnerdy F. R., Phan A. T. (2019). Angew. Chem., Int. Ed..

[cit55] Bakalar B., Heddi B., Schmitt E., Mechulam Y., Phan A. T. (2019). Angew. Chem., Int. Ed..

[cit56] Winnerdy F. R., Bakalar B., Maity A., Vandana J. J., Mechulam Y., Schmitt E., Phan A. T. (2019). Nucleic Acids Res..

[cit57] Do N. Q., Chung W. J., Truong T. H. A., Heddi B., Phan A. T. (2017). Nucleic Acids Res..

[cit58] Chung W. J., Heddi B., Schmitt E., Lim K. W., Mechulam Y., Phan A. T. (2015). Proc. Natl. Acad. Sci. U. S. A..

[cit59] Kettani A., Gorin A., Majumdar A., Hermann T., Skripkin E., Zhao H., Jones R., Patel D. J. (2000). J. Mol. Biol..

[cit60] Mishima Y., Kaizu H., Kominami R. (1997). J. Biol. Chem..

[cit61] Heller M., Flemington E., Kieff E., Deininger P. (1985). Mol. Cell. Biol..

[cit62] Matsugami A., Ouhashi K., Kanagawa M., Liu H., Kanagawa S., Uesugi S., Katahira M. (2001). J. Mol. Biol..

[cit63] Matsugami A., Okuizumi T., Uesugi S., Katahira M. (2003). J. Biol. Chem..

[cit64] Do N. Q., Lim K. W., Teo M. H., Heddi B., Phan A. T. (2011). Nucleic Acids Res..

[cit65] Haase L., Karg B., Weisz K. (2019). ChemBioChem.

[cit66] Li X. M., Zheng K. W., Zhang J. Y., Liu H. H., De He Y., Yuan B. F., Hao Y. H., Tan Z. (2015). Proc. Natl. Acad. Sci. U. S. A..

[cit67] Winnerdy F. R., Das P., Heddi B., Phan A. T. (2019). J. Am. Chem. Soc..

[cit68] De Y., Zheng He, K. W., Wen C. J., Li X. M., Gong J. Y., Hao Y. H., Zhao Y., Tan Z. (2020). J. Am. Chem. Soc..

[cit69] Li X. M., Zheng K. W., Hao Y. H., Tan Z. (2016). Angew. Chem., Int. Ed..

[cit70] Heddi B., Martín-Pintado N., Serimbetov Z., Kari T. M. A., Phan A. T. (2016). Nucleic Acids Res..

[cit71] Wang K. B., Dickerhoff J., Wu G., Yang D. (2020). J. Am. Chem. Soc..

[cit72] Phan A. T., Kuryavyi V., Gaw H. Y., Patel D. J. (2005). Nat. Chem. Biol..

[cit73] Jana J., Weisz K. (2020). Chem. – Eur. J..

[cit74] Phan A. T., Kuryavyi V., Burge S., Neidle S., Patel D. J. (2007). J. Am. Chem. Soc..

[cit75] Wei D., Parkinson G. N., Reszka A. P., Neidle S. (2012). Nucleic Acids Res..

[cit76] Pan B., Xiong Y., Shi K., Sundaralingam M. (2003). Structure.

[cit77] Pan B., Shi K., Sundaralingam M. (2006). Proc. Natl. Acad. Sci. U. S. A..

[cit78] Meier M., Moya-Torres A., Krahn N. J., McDougall M. D., Orriss G. L., McRae E. K. S., Booy E. P., McEleney K., Patel T. R., McKenna S. A., Stetefeld J. (2018). Nucleic Acids Res..

[cit79] Mukundan V. T., Do N. Q., Phan A. T. (2011). Nucleic Acids Res..

[cit80] Mukundan V. T., Phan A. T. (2013). J. Am. Chem. Soc..

[cit81] Martadinata H., Phan A. T. (2014). Biochemistry.

[cit82] De Nicola B., Lech C. J., Heddi B., Regmi S., Frasson I., Perrone R., Richter S. N., Phan A. T. (2016). Nucleic Acids Res..

[cit83] Sengar A., Vandana J. J., Chambers V. S., Di Antonio M., Winnerdy F. R., Balasubramanian S., Phan A. T. (2019). Nucleic Acids Res..

[cit84] Lenarčič Živković M., Rozman J., Plavec J. (2020). Molecules.

[cit85] Nguyen T. Q. N., Lim K. W., Phan A. T. (2020). Nucleic Acids Res..

[cit86] Gajarský M., Lenarčič Živković M., Stadlbauer P., Pagano B., Fiala R., Amato J., Tomáška L., Šponer J., Plavec J., Trantírek L. (2017). J. Am. Chem. Soc..

[cit87] Phan A. T., Kuryavyi V., Darnell J. C., Serganov A., Majumdar A., Ilin S., Raslin T., Polonskaia A., Chen C., Clain D., Darnell R. B., Patel D. J. (2011). Nat. Struct. Mol. Biol..

[cit88] Krishnan-Ghosh Y., Liu D., Balasubramanian S. (2004). J. Am. Chem. Soc..

[cit89] Marsh T. C., Henderson E. (1994). Biochemistry.

[cit90] Marsh T. C., Vesenka J., Henderson E. (1995). Nucleic Acids Res..

[cit91] Bose K., Lech C. J., Heddi B., Phan A. T. (2018). Nat. Commun..

[cit92] Webba da Silva M. (2003). Biochemistry.

[cit93] Webba da Silva M. (2005). Biochemistry.

[cit94] Ma’Ani Hessari N., Spindler L., Troha T., Lam W. C., Drevenšek-Olenik I., Webba Da Silva M. (2014). Chem. – Eur. J..

[cit95] Le H. T., Miller M. C., Buscaglia R., Dean W. L., Holt P. A., Chaires J. B., Trent J. O. (2012). Org. Biomol. Chem..

[cit96] Rauser V., Weinhold E. (2020). ChemBioChem.

[cit97] Zhang N., Gorin A., Majumdar A., Kettani A., Chernichenko N., Skripkin E., Patel D. J. (2001). J. Mol. Biol..

[cit98] Phan A. T., Kuryavyi V., Ma J.-B., Aurélie F., Andréola M.-L., Patel D. J. (2005). Proc. Natl. Acad. Sci. U. S. A..

[cit99] Phan A. T., Do N. Q. (2013). Nucleic Acids Res..

[cit100] Maity A., Winnerdy F. R., Chang W. D., Chen G., Phan A. T. (2020). Nucleic Acids Res..

[cit101] Adrian M., Ang D. J., Lech C. J., Heddi B., Nicolas A., Phan A. T. (2014). J. Am. Chem. Soc..

[cit102] Nielsen J. T., Arar K., Petersen M. (2009). Angew. Chem., Int. Ed..

[cit103] Haase L., Dickerhoff J., Weisz K. (2020). Chem. – Eur. J..

[cit104] Haase L., Weisz K. (2020). Chem. Commun..

[cit105] Haase L., Weisz K. (2020). Nucleic Acids Res..

[cit106] Liu Y., Lan W., Wang C., Cao C. (2018). J. Biol. Chem..

[cit107] Marušič M., Plavec J. (2019). Molecules.

[cit108] Kuryavyi V., Patel D. J. (2010). Structure.

[cit109] Črnugelj M., Šket P., Plavec J. (2003). J. Am. Chem. Soc..

[cit110] Wan C., Fu W., Jing H., Zhang N. (2019). Nucleic Acids Res..

[cit111] Tan D. J. Y., Das P., Winnerdy F. R., Lim K. W., Phan A. T. (2020). Chem. Commun..

[cit112] Nguyen T. Q. N., Lim K. W., Phan A. T. (2017). Sci. Rep..

[cit113] Asamitsu S., Obata S., Phan A. T., Hashiya K., Bando T., Sugiyama H. (2018). Chem. – Eur. J..

[cit114] Vianney Y. M., Preckwinkel P., Mohr S., Weisz K. (2020). Chem. – Eur. J..

